# Rational inattention in mice

**DOI:** 10.1126/sciadv.abj8935

**Published:** 2022-03-04

**Authors:** Nikola Grujic, Jeroen Brus, Denis Burdakov, Rafael Polania

**Affiliations:** 1 Institute for Neuroscience, Department of Health Sciences and Technology, ETH Zurich, Zurich, Switzerland.; 2 Neuroscience Center Zürich, Zurich, Switzerland.; 3 Decision Neuroscience Lab, Department of Health Sciences and Technology, ETH Zurich, Zurich, Switzerland.

## Abstract

Behavior exhibited by humans and other organisms is generally inconsistent and biased and, thus, is often labeled irrational. However, the origins of this seemingly suboptimal behavior remain elusive. We developed a behavioral task and normative framework to reveal how organisms should allocate their limited processing resources such that sensory precision and its related metabolic investment are balanced to guarantee maximal utility. We found that mice act as rational inattentive agents by adaptively allocating their sensory resources in a way that maximizes reward consumption in previously unexperienced stimulus-reward association environments. Unexpectedly, perception of commonly occurring stimuli was relatively imprecise; however, this apparent statistical fallacy implies “awareness” and efficient adaptation to their neurocognitive limitations. Arousal systems carry reward distribution information of sensory signals, and distributional reinforcement learning mechanisms regulate sensory precision via top-down normalization. These findings reveal how organisms efficiently perceive and adapt to previously unexperienced environmental contexts within the constraints imposed by neurobiology.

## Teaser

Computational models reveal that apparently irrational behavior in fact reflects efficient adaptation to biological limitations.

## INTRODUCTION

Seemingly irrational behavior is unexpectedly common in humans and other animals. This has been widely documented in research conducted by neurobiologists, psychologists, and economists, for whom apparent anomalies in the behavior of healthy organisms are difficult to reconcile with idealized statistical and neurobiological frameworks (*1*, *2*). These idealized concepts and theories are increasingly used to guide diagnoses and treatments in the medical domain (*3*) and policy-making in applied economic settings (*4*), so these anomalies raise an important question: Why are common behavioral strategies so often different from the idealized predictions, and is it reasonable to dismiss such strategies as irrational or suboptimal?

A potential answer to this question might be rooted in a premise that holds across all living organisms: Organisms have a limited metabolic budget for interacting with their environments, and thus a restricted capacity to process environmental and interoceptive signals (*5*). This entails that, for instance, when an organism must decide between various alternatives that promise some reward, the process of choosing the best alternative must be based on imprecise and biased perceptions (*6*). However, it remains poorly understood how organisms can make the best out of these perceptual limitations such that reward is maximized in environments that are uncertain.

One attempt to address this question proposes that, in the course of evolution, the nervous system developed computational strategies that take into consideration the statistical structure of the environment and the uncertainty of sensory signals. For instance, theoretical (*7*), behavioral (*8*, *9*), and recent neurophysiological (*10*) studies support the idea that the brain learns a statistical model of the world and optimally combines this knowledge with imperfect sensory information and thus approaches optimal computations under uncertainty. However, this approach does not take into consideration the biological limitations of information processing in the nervous system.

This problem has received considerable attention in recent years, when the principles of efficient computation under uncertainty and cognitive limitations can be studied in the rational inattention or resource rationality framework (*11*, *12*). This approach has been instrumental in accounting for various aspects of behavior that were not possible to explain with rational models, not only in decisions relying on basic sensory perception (*13*) and memory (*14*) but also in higher-level processes in humans such as the evolution of language (*15*) and economic decision-making (*16*–*19*).

Rational inattention theory predicts that (i) limited time, (ii) noise in the system and environment, and (iii) metabolic constraints all limit access to information. Consequently, a decision-maker may often benefit from violating the axioms of rational modeling frameworks (*20*) by being rationally myopic and ignoring information that is not worth the effort of processing (*21*, *22*). These apparently suboptimal strategies may ultimately lead organisms to maximize their chances of survival (*23*, *24*).

Nonetheless, formulations of rational inattention, which have, to date, been applied predominantly in economic settings, often do not display close conformity with neurobiological implementations. Moreover, in some cases, these formulations may not be directly translatable to applications of optimal allocation of attentional resources in sensory perception (*25*). In addition, previous formulations leave unclear how organisms efficiently adapt and exploit previously unexperienced stimulus-reward associations when sensory information varies in its reliability. In other words, what computations can allow organisms to allocate their limited neural resources in an adaptive manner on the basis of experience of reward and punishment? Here, we developed a general modeling framework to study how organisms endogenously allocate their attention with limited resources such that reward consumption of the organism is maximized when it interacts with the environment under uncertainty.

Formal tests of rational inattention have been predominantly tested in humans, and it remains unknown whether these influential concepts can be tested and also hold in lower-order species such as mice. Should this be the case, the availability of genetic tools and large-scale cellular imaging in mice would enable the concepts studied in this work to be used to address various open questions about limited information processing capacity (*26*) in large-scale experimental settings. This would accelerate the translation from neurobiological mechanisms to formal concepts of rational inattentive behavior in medical settings and applied economics.

To test the neurobehavioral underpinnings of rational inattention, we studied whether mice could be trained to perform a choice task requiring them to make decisions on the basis of ordinal comparisons of orientation stimuli spanning the whole sensory space. This task decouples decisions from absolute sensory information contained in each individual decision alternative, rendering only measures of relative information relevant. This is essential to consider given that, in ecologically valid settings, organisms typically encounter situations in which abstract choices are invariant to specific visual stimuli; for instance, when choosing between stimuli A = 1 and B = 2 drops of juice, the mouse must choose B, but between B = 2 and C = 4, the mouse must choose C and not B. In addition, we introduced trial-to-trial variability in the reliability of sensory stimuli and controlled the prior distribution of the sensory inputs such that it matched innate and possibly evolutionary preserved neural sensory codes in mice. During its experimental lifetime, each animal experienced previously unexperienced stimulus-reward association rules in different environments, while the physical statistics of the environment and task structure remained identical. Controlling and manipulating all these components in the decision task provide a unique opportunity to study whether and how mice learn to adapt their sensory processing resources to maximize reward outcome in each environment without any cues other than trial-by-trial experience.

We found that mice behave as rational inattentive agents by taking into consideration their information processing limitations to develop sensory encoding strategies that lead them to maximize reward consumption. This suggests that many aspects of variable and apparently irrational behavior both in the perceptual and economic domain reflect the efficient use of a limited metabolic budget to operate and interact with the environment.

## RESULTS

### Behavioral task and behavior

**Fig. 1.**
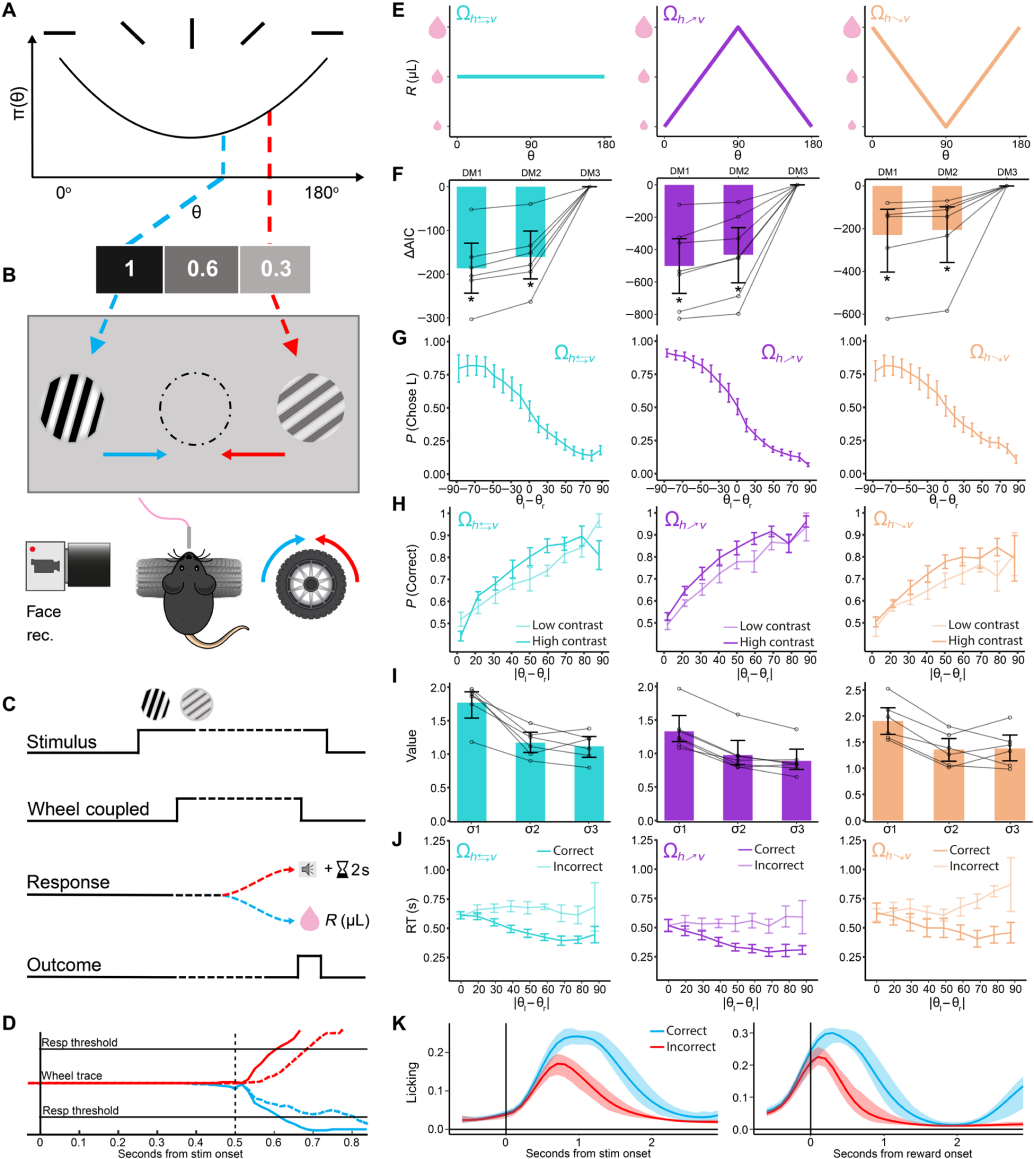
Paradigm and behavior. (**A**) Two orientations were drawn from a prior distribution with a given contrast level. (**B**) The animal turns the wheel to move the more vertical grating to the middle of the screen to obtain a milkshake reward. (**C**) Trial timeline and outcomes. (**D**) Median wheel trace for correct (full line) and incorrect (dashed line) trials. Trials picking the right side are plotted in red, and those picking the left side are in blue. (**E**) The three stimulus-reward mapping conditions Ω. (**F**) Comparison of Akaike information criterion (AICs) for the three descriptive models (DMs) and the three reward mappings relative to DM3. Individual mice are shown with semitransparent gray lines. Error bars represent bootstrapped 95% confidence interval (CI) of the mean across mice. *Bootstrapped 95% CI AIC difference is below zero. (**G**) Psychometric curves. Angle difference, as marked on the *x* axis, is the difference of absolute value of each side angle minus 90°. (**H**) Percentage correct across difficulties for low-contrast trials (semitransparent; sum contrasts <1) and high-contrast trials (solid; sum contrasts >1). (**I**) Perceptual noise to each contrast level in DM3 (σ1, σ2, and σ3 for contrast values 0.3, 0.6, and 1, respectively). Individual mouse weights plotted as semitransparent lines. (**J**) Mean reaction times across difficulties for correct (solid lines) and incorrect trials (semitransparent lines). In (G to J), error bars represent SEMs. (**K**) Extracted licking rate aligned to stimulus onset (left) and reward onset (right) for correct (blue) and incorrect (red) trials (shaded area represents SEM).

We trained mice (*n* = 7) in a two-alternative forced choice task (2AFC), in which they were presented two gratings θ_l_ and θ_r_ (presented on the left and right side of the screen, respectively) and had to choose the one that was more vertically oriented by turning the wheel under their forepaws and thereby moving the grating to the center of the screen (Fig. 1, A to D, and Methods). The two alternatives θ_l_ and θ_r_ are independently drawn from a prior distribution π(θ) (Fig. 1, A and B; see below). To study the role of sensory uncertainty, we randomly varied the contrast levels for each of the two stimuli across different levels on a trial-to-trial basis (Fig. 1B; see Methods for details). In the following, we first describe how we derived the prior π(θ) used in our study, then proceed to explain the experimental paradigm of the decision-making task, and present the descriptive behavioral results.

### Prior distribution approximation of stimulus orientation in mouse V1

To specify the prior distribution π(θ) used in our task, we investigated whether it would be possible to estimate the innate information coding of orientations in the mouse primary visual cortex (V1). To this end, we relied on a large-scale cellular imaging dataset based on simultaneous recordings from ∼20,000 neurons in mouse V1, while static gratings at a random orientation were presented on each trial (*27*). Here, we paid particular attention to two pieces of information: (i) the distribution of preferred orientations across V1 cells and (ii) the neural decoding orientation error of a linear decoder. Regarding the distribution of orientations, the data show that, in mouse V1, a larger proportion of neurons are dedicated to code horizontal relative to vertical orientations for static stimuli (fig. S1A). On the basis of this result, theories of efficient neural coding predict the following: (i) Decoding error should be larger for orientations with fewer neural resources, thus higher decoding error for vertical orientations in mice, and (ii) this decoding error shape across the sensory space should be amplified for shorter exposure to sensory stimulation (*13*). In line with these predictions, the data show that decoding error was higher for vertical than horizontal orientations, and this pattern was further amplified in the condition with shorter stimulus presentation (fig. S1B). These results confirm that mice appear to dedicate more neural resources to encode horizontal relative to vertical orientations. We speculate that this prior distribution could have been induced in mice by evolution (or perhaps life experience) because of their anatomy and morphology. However, this notion would need to be more formally tested in future studies, for instance, as previously studied in cats, a species that appears to be more exposed to horizontal than vertical orientations in their natural environment (*28*).

On the basis of these analyses, here, we specified the distribution of orientations π(θ) such that horizontal angles were more common than vertical angles (Fig. 1A). This allowed us to focus our analyses on studying how mice adopt reward-maximizing coding strategies based on specific reward-stimulus contingencies while preserving prior neural codes that appear to be innately present in mouse early visual areas.

### Mice can perform ordinal comparisons under uncertainty in a continuous sensory space

In the 2AFC task, orientations of the two gratings were drawn from distribution π(θ) (Fig. 1A). For all conditions tested in this study, the mice had to choose the grating that was more vertically oriented and received a reward *R* (a given amount of milkshake) if and only if a correct decision was made. Rewards were based on three different stimulus-reward association environments Ω (Fig. 1E and table S1). After the mice reached stable behavior in a baseline decision task, they performed between 20 and 50 sessions in each environment Ω (Methods).

In environment Ω_*R*:*h*⇄*v*_, mice received a fixed amount of milkshake for correct decisions irrespective of orientation (Fig. 1E, left). This condition corresponds to the reward contingencies classically implemented in perceptual decision-making tasks. In environment Ω_*R*:*_h_*↗*^v^*_, more vertical orientations yielded higher rewards for correct decisions via a linear relationship (Fig. 1E, middle). Last, in environment Ω_*R*:*^h^*↘*_v_*_, more horizontal orientations yielded a higher reward (Fig. 1E, right). Recall that, in all cases, the decision rule is to choose the orientation that is more vertical. Thus, counterintuitively, in any given trial in environment Ω_*R*:*^h^*↘*_v_*_, mice should choose the orientation that is mapped to the smallest reward because the other option is less vertical and therefore leads to no reward at all.

First, we investigated on the basis of simple descriptive models (DMs) whether mice were able to follow the basic decision rule in each environment Ω (all models incorporated lapse rates and trial history effects; Methods). Using a simple DM (DM1) incorporating the angle difference Δθ between the input stimuli in a given trial, we found that, for all environments Ω, mice learned to choose the more vertical orientation (slope of psychometric curve; logistic mixed effects *P* < 0.001 for all Ω; Fig. 1G and fig. S1). This indicates that, during their decisions, the abstract choice rule was decoupled from specific sensory information, and relative information was the relevant variable. These results are, in principle, in accordance with a recent study also showing that mice can apply this sort of abstract decision rules (*29*).

In a second model (DM2), we investigated whether stimulus contrast affected mice decisions. As expected, we found that the lower the contrast, the lower the sensory sensitivity of mice (mixed effects model; effect of contrast on choice accuracy *P* < 0.001 for all environments Ω; Fig. 1, I and H). In a third model, we investigated whether, in addition to the influence of stimulus contrast on decision noise, the difference in stimulus contrast between the two orientations affected mice decisions (DM3). In addition to the impact of angle difference and contrast on decision-making, we found that mice had a preference to choose the orientation with higher-contrast stimulus (mixed effects model; *P* < 0.001 for all Ω). This model explained the data better than the simpler models for all mice and all environments [bootstrap 95% confidence intervals (CIs) of AIC_DM1_ − AIC_DM3_ < 0 and AIC_DM2_ − AIC_DM3_ < 0 for all Ω; Fig. 1F]. While, by design, contrast should not play a relevant role in mice decisions, below, we will show that an optimal observer model can partially account for this bias (see below). We speculate that the remaining unexplained variance might be related to spillovers of mice being driven to perform a detection task. We found that lapse rates in DM3 were relatively low (λ = 0.091, 95% CI[0.044, 0.14]), suggesting that mice were engaged and followed the decision rules of our task. Nevertheless, we also explored models that additionally included separate lapse rates (DM4), reward size in the previous trial (DM5), and both of these additional parameters (DM6; see Methods). We found that none of these additional models decisively improved data quantification. Table S2 contains an overview of the parameters fitted to the data in all the models described above.

For completeness, we investigated whether reaction times (RTs) show the behavioral patterns often observed in both perceptual and value-based decision tasks as a function of absolute evidence and trial correctness. We found that mice RTs were faster for correct responses (Fig. 1J; see fig. S2 for individual mouse data). In addition, the higher the absolute evidence (Δθ), the faster the response for correct decisions (mixed linear model; *P* < 0.001 for all Ω). These results are in line with chronometric behavior reported across species both in perceptual and value-based decisions (*30*–*32*).

Together, the behavior exhibited by the mice in our study indicates that they can learn to perform decision tasks that decouple decisions from sensory information, where measures of an abstract decision rule based on relative information are relevant. Next, our goal is to investigate what neurocomputational mechanisms might guide behavior under cognitive limitations.

### Efficient perception and rational inattention

**Fig. 2.**
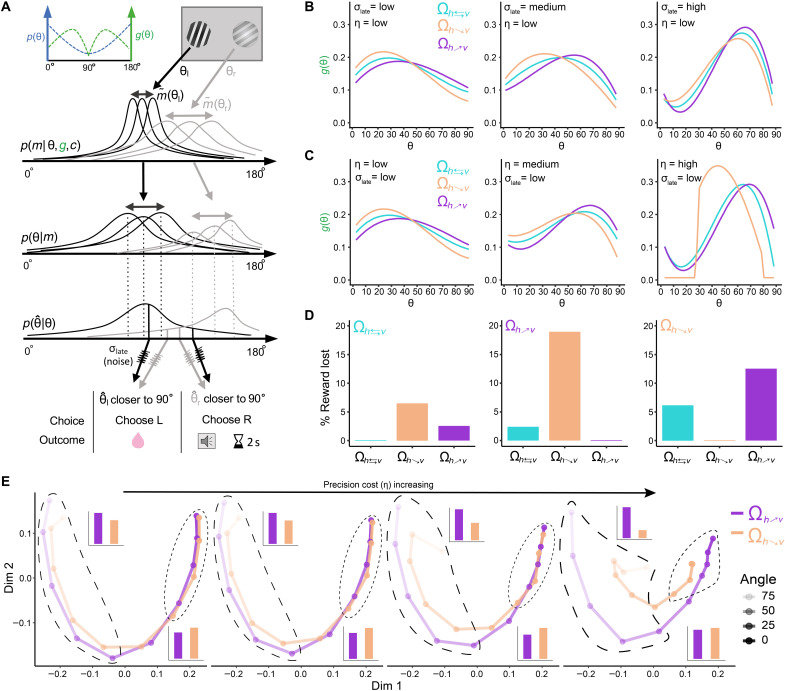
Rational inattention model and predictions. (**A**) Top: Prior distribution of orientations (blue) and illustration of *g* function (green). Middle: Illustration of the inference model. A noisy measurement *m* is obtained for each stimulus θ according to the likelihood function *p*(*m*∣θ,·). Then, prior and likelihood function information are combined to derive a posterior distribution. Subsequently, the observer computes a posterior estimate $\hat{\theta }$ [generating a posterior of estimates $p(\hat{\theta }|\theta )$], which can be corrupted by late noise σ_late_. Bottom: Table of choices/outcomes dependent on noisy decoding. (**B** and **C**) Optimal solutions for *g* at three reward mappings and noise levels. The resource gain functions *g* that guarantee maximal reward consumption depend on the levels of both encoding cost η and late noise σ_late_. (**D**) Example of percent reward loss when optimal solutions for each environment Ω are swapped for another one. (**E**) Geometric analysis of psychometric performance based on multidimensional scaling (MDS). Geometric distance between a pair of stimuli (each represented by a point) represents the degree of discriminability. We present the MDS for environments Ω_*R*:*_h_*↗*^v^*_ and Ω_*R*:*^h^*↘*_v_*_ at different levels of sensory precision cost η. Barplot insets show that, in general, average geometric distance for angles closer to vertical is larger (top left and long dashed lines) and smaller for horizontal (bottom right and short dashed lines) in environment Ω_*R*:*_h_*↗*^v^*_.

We developed a framework, based on principles of optimal statistical inference, that allowed us to study how sensory systems should allocate coding resources under the assumption that there is a limit in information processing at two stages of the decision process: (i) limited precision during sensory encoding and (ii) limited precision in downstream decision circuits. Crucially, given that the physical prior π(θ) remains constant, these predictions may be specific to the stimulus-reward associations of a given context or environment Ω. Here, it is assumed at all times that the goal of the organism is to maximize reward consumption (Fig. 2A; see Methods for a detailed specification of the model).

Given that noisy communication channels always lose information during transmission, we argue that it is more efficient for the brain to adapt to the reward-maximizing rules of a particular environment at the earliest stages of sensory processing. This notion is supported by evidence suggesting that early sensory systems represent not only information about physical sensory inputs but also nonsensory information according to requirements of a specific task and the behavioral relevance of the stimuli (*33*, *34*). Thus, a key assumption of our framework is that, despite the fact that the physical prior distribution was held constant for all environments Ω, sensory codes adapt to environmental demands, possibly via feedback schemes (*35*).

Another key assumption that we make in our framework is that organisms do not have unlimited neural resources that can be dedicated to guide behavior. More specifically, in our study, limited coding resources in sensory systems must be optimally allocated given bounded metabolic resources and physiological constrains that do not allow the system to be nearly infinitely precise in processing information at all segments of the sensory space. Thus, we assume that precise information coding comes at some cost. More formally and briefly, we assume that, in every trial, a mouse obtains a noisy measurement *m* independently for each input orientation θ. We model the noisy measurements using the von Mises distribution

$$p(m;\theta ,c,g)\propto e^{k(c)g(\theta )\text{cos}(\theta -m)}$$ (1)

where the precision of the measurement is determined by two multiplicative factors: (i) *k*(*c*), which determines the degree of sensory encoding precision at given contrast level *c*. Theoretically, *k*(*c*) could be infinitely large, but the costs per precision unit mentioned above suggest that sensory precision as a function of contrast must be a bounded quantity. (ii) A resource gain function *g*(θ), which is a normalized function that determines how the bounded resources should be allocated over the whole sensory space. By definition, this term is bounded and acts as a resource distributor. Last, sensory encoding precision for a given angle input θ and contrast *c* is given by the multiplicative factor *k*(*c*)*g*(θ) (Methods).

On each trial, the agent computes a posterior distribution *p*(θ∣*m*) by combining the physical environmental prior distribution π(θ) with the likelihood of the measurement *p*(*m*∣θ) via Bayes rule. Then, the agent applies a decoding rule to obtain a posterior estimate $\hat{\theta }$, which we assume to be the expected value of the posterior distribution *E*[*p*(θ∣*m*)], also known as Bayesian least squares (BLS) estimator. We assume that, on each trial, the mouse independently estimates ${\hat{\theta }}_{l}$ and ${\hat{\theta }}_{r}$ for the input orientations θ_l_ and θ_r_, respectively, and then makes a decision on the basis of the abstract decision rule in our task: Choose the more vertical angle.

If we constrain *g*(θ) to be a positive and normalized function and assume a cost *K* associated to achieve a certain level of precision during sensory encoding (*5*), then one can formulate a well-constrained optimization problem to find the optimal allocation of resources in the orientation space for (i) a given physical environmental prior π(θ), (ii) a characteristic contrast response function *k*(*c*), (iii) reward outcomes associated to decision-outcome rules in a given context or environment Ω, and (iv) downstream noise σ_late_ that is not related to sensory encoding. Formally, the goal is to find a resource gain function g* and the maximum allowed precision/neural activity *k*_max_ [with an underlying contrast response function *k*(*c*); see Methods] such that

$$\underset{g,k_{\text{max}}}{\text{max}}\;E[\text{reward}|\Omega ,k(c),\sigma_{\text{late}},\pi ]-K(\bar{k},\eta )$$ (2)

where *c* is the set of contrasts that the animal experiences. We model the cost *K* as a function of the average precision $\bar{k}$ that the agent invests on solving the inference problem, where η > 0 indicates how much the metabolic cost scales with average precision $\bar{k}$ (Methods). We argue that this choice of cost function is reasonable when applied to the visual system, given that, in our model, sensory encoding precision (Eq. 1) can be directly related to the amount of neural activity and its associated variability (Methods) (*9*). Moreover, it has been recently shown that the relationship between neural activity and energy expenditure in the brain is nearly linear (*36*).

Here, we highlight that an important feature of the rational inattention framework is that allocation of sensory precision [*g*(θ) and *k*_max_] does not need to be assumed or manually fitted, as it is often the case in plain Bayesian frameworks, but emerge endogenously by assuming a cost per precision unit η and downstream precision σ_late_, alongside the associated (reward) loss that the organism experiences (Methods).

### Optimal resource allocation depends on both sensory encoding precision and downstream noise

We studied whether the optimal resource gain function *g* depended on the amount sensory precision cost η. In addition, we also studied the dependence of *g* on sources of downstream noise σ_late_, as recent evidence suggests that limits of sensory perception in mice might be also related to downstream neurocomputational imprecision (*27*), possibly related to computational limitations of downstream decoders and other forms of irreducible noise in the system (*37*). Here, we would like to emphasize that a key feature of our study is that, for all three different stimulus-reward environments Ω, the physical prior π(θ) was always the same, and therefore, potential differences in the resource gain function *g*(θ) must be related to internally improving sensory representations of the learned associations that maximize reward expectation. To derive the optimal *g* functions for a particular combination of η and σ_late_, we used the exact distribution of stimuli inputs used in the mice experiments.

We found that, in general, for low levels of downstream noise σ_late_ and relatively low costs in encoding precision η, more resources should be allocated to segments of the orientation space with higher prior π(θ) density (Fig. 2B, left, and figs. S3 and S4). This result is in line with previous studies suggesting that, in the limit of low sensory noise (i.e., η → 0), encoding precision should be higher for segments of the orientation space with more density (*13*, *38*). For instance, it can be shown that, for the case of perceptual tasks with constant reward delivery per correct trial (i.e., Ω_*R*:*h*⇄*v*_ environment in our study), discrimination thresholds are inversely proportional to the prior distribution but crucially in the low-noise regime (*13*). However and unexpectedly, as σ_late_ increases, the predictions of these analytical solutions breakdown, and agents should start to be myopic to segments of the sensory space where the prior’s density is high (Fig. 2B, middle and right). Similarly, assuming no downstream noise (i.e., σ_late_ = 0), instead, varying the levels of sensory noise reveals a similar but qualitatively different pattern of optimal resource allocation strategies as a function of sensory precision costs (Fig. 2C and figs. S3 to S5). Thus, these predictions reveal two important features of the rational inattentive agent. First, optimal resource allocation does depend not only on the level of encoding precision of sensory signals but also on stimulus independent downstream noise. Second, long-held conceptions that neural resources should always be allocated to spaces with higher prior density do not necessarily hold beyond the low-noise regime.

Why are more resources allocated away from high prior density spaces when precision cost and downstream noise are relatively high? On the one hand, for the case of high-precision cost, low levels of encoding noise generate higher levels of Bayesian attractive biases. Thus, optimal solutions of the rational inattention model reveal that if the system has the opportunity to flexibly modulate its sensory gain, then it pays off to put more weight at places of the sensory space that avoid poor discriminability due to overall attractive biases. To provide a better intuition behind this result, we carried out geometric analyses of psychophysical performance based on multidimensional scaling (MDS) (Fig. 2E and fig. S6). MDS reveals that the effect of attractive biases for high precision costs is compensated by expanding the discriminability of orientations located at intermediate levels of verticality. On the other hand, for the case of high downstream noise, augmenting precision at the edges of this space does not increase the discriminability of neighboring orientations beyond the most vertical and most horizontal orientation. That is why, for relatively high levels of noise, there is preference to allocate more attentional resources to rather intermediate levels of cardinality (Fig. 2E and fig. S6).

While this pattern is similar for all environments Ω considered here for a given combination of η and σ_late_ (Fig. 2, B and C, and fig. S3), there are important differences in the resource gain functions *g* between environments. We found that resource allocation has a tendency to be relatively higher for more vertical angles in environment Ω_*R*:*_h_*↗*^v^*_ (Fig. 1E, middle); however, this requires sacrificing precision at more horizontal orientations. This pattern reverses for environment Ω_*R*:*^h^*↘*_v_*_. This result intuitively makes sense, given that, in environment Ω_*R*:*_h_*↗*^v^*_, more vertical angles deliver more reward, and therefore, mice are better off at giving up encoding precision for horizontal orientations even if they occur more often. Another key prediction of the rational inattention framework is that, for environment Ω_*R*:*_h_*↗*^v^*_, the system should allocate more resources to solve the decision task relative to Ω_*R*:*^h^*↘*_v_*_ (this is evident in the MDS analyses where nodes of the manifold are more separated in Ω_*R*:*_h_*↗*^v^*_ relative to Ω_*R*:*^h^*↘*_v_*_). This prediction emerges in the rational inattention framework because the relative allocation of resources under our inference framework leads to slightly higher reward expectation in environment Ω_*R*:*^h^*↘*_v_*_ relative to Ω_*R*:*_h_*↗*^v^*_. We also considered rational inattentive model that does not allow a variable and adaptive resource gain function *g*, but instead, we assume it to be constant. While this model captures the effect where, in environment Ω_*R*:*_h_*↗*^v^*_, the system allocates more resources to solve decision task relative to Ω_*R*:*^h^*↘*_v_*_, this model does not account for trade-offs in discriminability between horizontal and vertical spaces across environments (fig. S7).

Last, we investigated what is the actual benefit of applying the most efficient resource allocation in a given environment. Using average parameters of model fits to the mice data (see below), we found that agents could lose around 10 to 20% of the reward if resources are not optimally allocated (Fig. 2D). This confirms that efficient resource allocation under uncertainty and cognitive limitations is critical to maximize reward.

### Adaptive rational inattention in mice

**Fig. 3.**
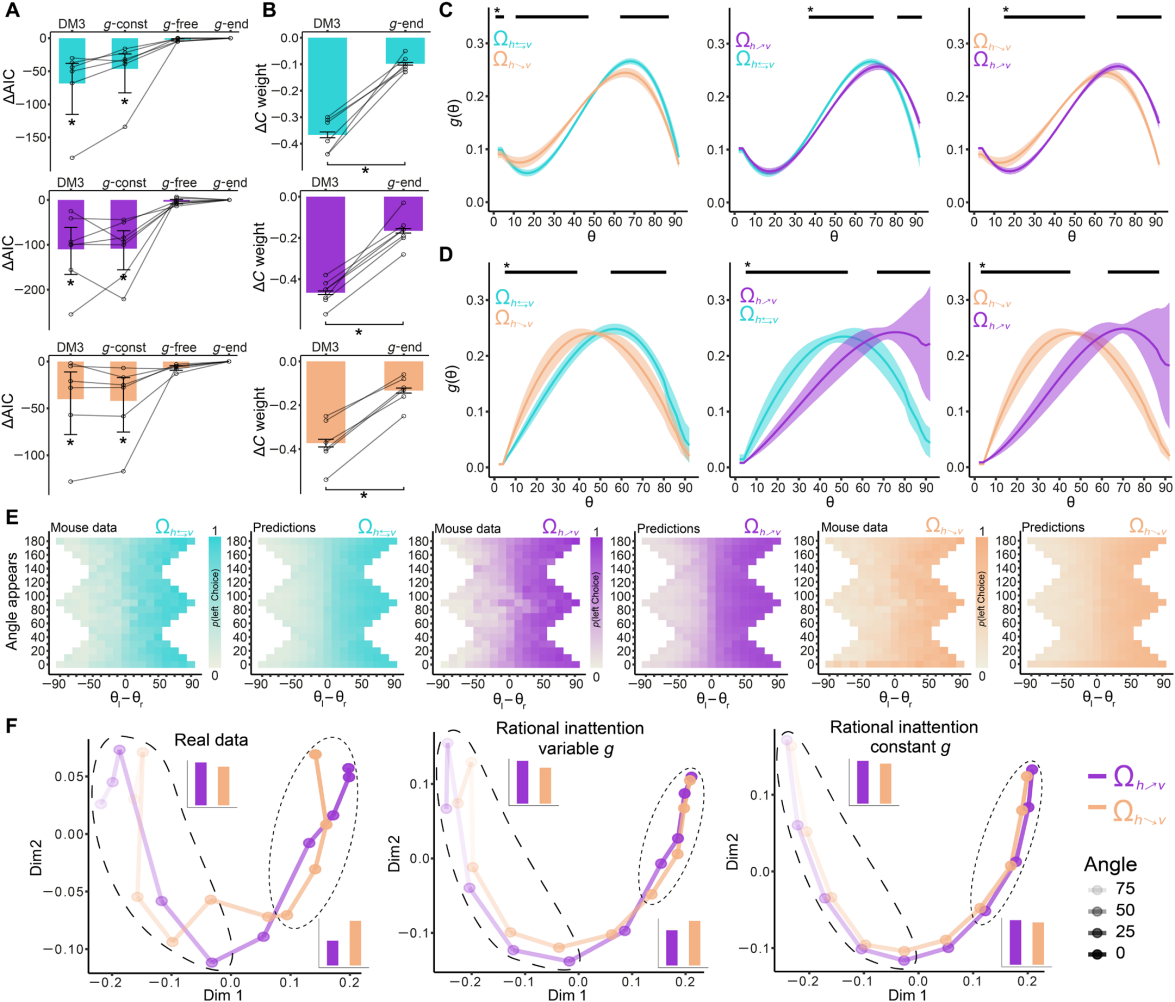
Empirical rational inattention results. (**A**) The rational inattention model provides the best account of the data. The figure shows the difference in AIC relative to the *g*-endog model (*g*-end). Error bars represent the bootstrap 95% CI across mice, and individual mice are shown with transparent lines. *Bootstrap 95% CI significant difference. (**B**) Probit weights capturing the influence of contrast difference between the two stimuli on choice. Preference to choose the higher-contrast stimulus is largely captured by the rational inattention model. Error bars represent SEMs, and individual mice are marked by transparent lines. *P < 0.001, significant differences mixed effects model. (**C** and **D**) Pairwise comparisons of resource allocation functions *g*(θ) for each reward environment. Black lines signal significant differences, as determined by bootstrap 95% CIs of within mouse differences. (C) Results for *g*-endog model and (D) the non-endogenized model. See fig. S12 for individual mice data. (**E**) Heatmaps showing probability of choosing the left stimulus at each possible orientation difference (trial difficulty). Data and model predictions show overall good agreement. See fig. S9 for data at different contrast levels. (**F**) MDS analyses of real data and model fits reveal that the rational inattention model allowing adaptive *g* across the sensory space has a psychophysical geometry that closely resembles the real data. Barplots show that, for both real data and *g*-endog, average geometric distance for angles closer to vertical is larger (top left and long dashed lines) and smaller for horizontal (bottom right and short dashed lines) in environment Ω_*R*:*_h_*↗*^v^*_. These patterns are absent in the constant *g* model.

First, we investigated whether the rational inattention model that endogenizes *g*(θ) (*g*-endog model; see Methods) provides a better account of the mice data than the DMs. We found that the *g*-endog model provides better fits to the data for all environments (Fig. 3A and fig. S8). Notably, the rational inattention model has the same number of parameters as DM3. Therefore, the quantitative differences in goodness of fit between the two models are not related to model complexity (Fig. 3A). Inspection of the *g* functions across mice for each environment follows the counterintuitive qualitative predictions of the theory for non-negligible sensory and late noise in our task: More resources are allocated to angles close to vertical relative to horizontal orientations (Fig. 3C). In addition, more resources are allocated to vertical orientations in environment Ω_*R*:*_h_*↗*^v^*_ (higher reward for more vertical angles; Fig. 1E) relative to the other environments, while the opposite is the case for Ω_*R*:*^h^*↘*_v_*_ environment (cluster-corrected *P* < 0.05; Fig. 3C). These results strongly suggest that the manner in which mice allocate attention in light of their limited coding resources, in the presence of encoding and precision noise, follows the signatures of the rational inattention model. In addition, we found that the levels of contrast bias observed in the DM (DM3; see above) can be largely explained by the rational inattention model (Fig. 3B).

To make sure that the shapes and differences in resource gain function *g* are not a product of the endogenous solutions of the optimal models, we fitted a version of our inference model that does not endogenously restrict the shape of *g* but is fit in a model-free fashion (Methods). This model reveals two notable results: First, the qualitative shape of the resource gain function *g* across environments is similar to the optimal solutions [more resources are allocated away from segments of the sensory space with high prior π(θ) density]. Second, mice allocated their limited coding resources in a way that resembled the signatures of reward maximization in each environment Ω. More specifically, we found that, (i) in environment Ω_*R*:*_h_*↗*^v^*_ (more reward for more vertical angles), resource allocation was relatively higher for vertical angles, (ii) this pattern reversed for Ω_*R*:*^h^*↘*_v_*_, and (iii) resource allocation in environment Ω_*R*:*h*⇄*v*_ was located in between the other environments (cluster-corrected *P* < 0.05; see pairwise comparisons in Fig. 3D). Crucially, model comparison between the *g*-endog model and the *g*-free model reveals that the complexity of freely capturing the shape of resource allocation *g* is not necessary (bootstrap 95% CI of AIC_*g*-endog_ −AIC_*g*-free_ ≈0 for all Ω; Fig. 3A).

In addition to these quantitative results, our model also accounted for various qualitative features of mice behavior (Fig. 3, E and F, and fig. S9). The MDS analyses reveal that the *g*-endog model closely resembles the geometry of psychophysical performance of the real data (Fig. 3F). Here, two key predictions of the rational inattention model are observed in the behavioral data: (i) Geometric distance of the nodes in the MDS manifold at horizontal orientations is shorter for environment Ω_*R*:*_h_*↗*^v^*_ relative to Ω_*R*:*^h^*↘*_v_*_, and (ii) geometric distance is larger for environment Ω_*R*:*_h_*↗*^v^*_ relative to Ω_*R*:*^h^*↘*_v_*_ (Figs. 2E and 3F). Another prediction of the rational inattention model is that, for moderate levels of sensory and downstream noise (as is the case in mice), the general levels of discriminability should be larger in environment Ω_*R*:*_h_*↗*^v^*_ relative to Ω_*R*:*^h^*↘*_v_*_ (Fig. 2E and fig. S6). Analyses of sensory precision in the best DM (thus independent of the theory) reveal that σ(*c*) was higher for all contrasts in Ω_*R*:*_h_*↗*^v^*_ (bootstrap 95% CI of Δσ < 0 for Ω_*R*:*_h_*↗*^v^*_ relative to Ω_*R*:*^h^*↘*_v_*_). These results suggest that mice adopt efficient rational-inattentive perception according to the reward contingencies of a given environment.

Last, we compared the results of the *g*-endog model with an ideal observer model in which resource allocation was uniform across the whole sensory space (*g*-const model). We found that the *g*-endog model accounted better for the data than the *g*-const model for all mice and all environments Ω (bootstrap 95% CI AIC_*g*-endog_ −AIC_*g*-const_ <0 for all Ω; Fig. 3, A and F). These results strongly suggest that assumptions in ideal observer models where uniform likelihood functions are adopted (*39*), mainly for mathematical convenience, might not always be warranted.

### Adaptive rational inattention via reinforcement learning

**Fig. 4.**
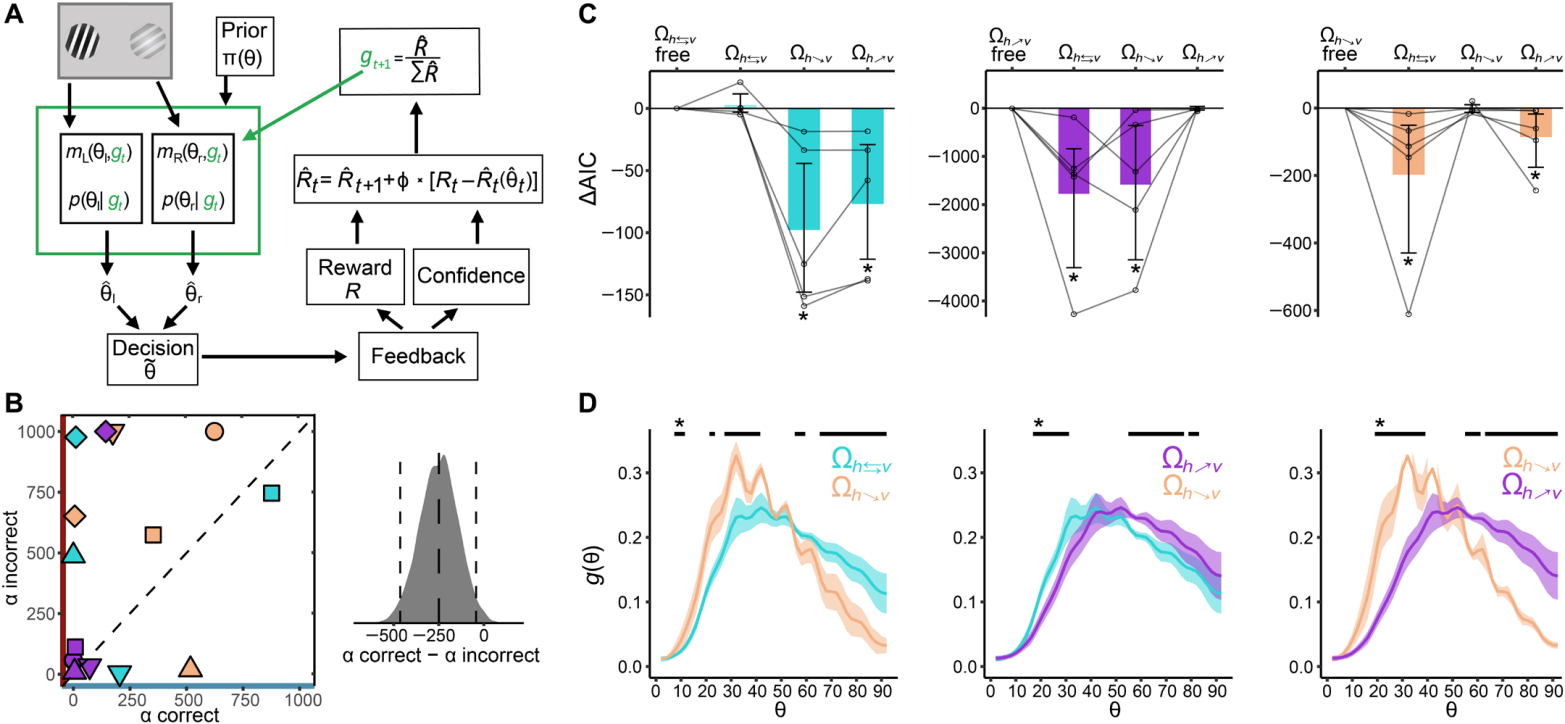
RL model. (**A**) Sketch of the RL model. Briefly, after the observer chooses an angle $\tilde{\theta }$, the resource gain function *g* is updated following a divisive normalization operation that depends on the reward received *R*, the level of confidence in the decision, and a distributional update of the reward distribution vector $\hat{R}$. (**B**) Left: Learning weights α for correct and incorrect trials. Individual mice are shown with different symbols and color-coded for condition (see Fig. 1D). Right: Distribution of bootstrapped differences of α for correct and incorrect trials; dashed lines show the mean and bootstrapped 95% CIs. Recall that, in our model, the learning rates α are divisive, and therefore, lower values indicate higher learning rates. (**C**) AIC difference between the *g*-endog model and the learning model for each environment Ω. Learning parameters are in each subplot from left to right swapped for each condition that shows that there were no differences between the endogenized rational inattention model and the learning model in its corresponding environment Ω. Semitransparent lines show the AIC differences for each mouse, and error bars represent the bootstrapped 95% CI across mice. *Bootstrap 95% CI significance. (**D**) Resource gain functions *g* from the RL model averaged for the last 500 trials in each environment Ω for each mouse and then averaged across mice. Error bars represent SEMs. Black lines on top show significant differences based on bootstrapped 95% CIs of within-subject differences relative to zero.

The rational inattention model implemented above assumes that mice have already adapted to a permanently relevant environment Ω. Hence, the optimal resource gain function *g* remains fixed across trials in a given environment. Therefore, this model does not explain how mice may reallocate their limited coding resources via trial-to-trial experience. To incorporate this fundamental aspect of behavior in our model, we implemented a reinforcement learning (RL) mechanism, allowing dynamical updating of the resource gain function *g* using operations that appear to be supported by biological neural systems (Fig. 4A; see Methods for details). Briefly, we assume that the animal updates, on the basis of experience, a distribution of rewards associated to sensory information, for instance, in the ventral tegmental area (VTA) (*40*), or any other downstream circuit performing similar operations. After a mouse makes a decision, the animal updates the reward distribution using a form of distributional updating (see Methods). Crucially, the strength of update (i.e., learning rate) is dynamically adjusted according to the confidence level of the decision just made (*41*). In addition, on the basis of compelling evidence suggesting that humans learn differently from positive and negative outcomes (*42*), we investigated whether our mice would show a similar behavior in our task by allowing separate learning rates for correct and incorrect decisions. In the final step, we assume that the brain readjusts the resource gain function *g* (possibly in early sensory areas) via a top-down divisive normalization operation (*43*).

If it is the case that the RL model described above converges to the static rational inattention model, then we expect a similar allocation of resource gain function *g* across environments Ω for both models. We found that the average resource gain functions *g* over time showed nearly identical patterns of those found in the static ideal observer model (Fig. 3D). Moreover, we found that the set of RL parameters that were fit to each condition improved model fits specifically for the corresponding environment Ω (Fig. 4C). This suggests that parameters controlling the dynamics of reward-sensory learning are specific to the reward-maximizing rules of a given context. In addition, the difference between the static rational inattention and RL models was not distinguishable across mice for all environments Ω (ΔAIC(Ω_*R*:*h*⇄*v*_) = 2.8[−3.4,12.4]; ΔAIC(Ω_*R*:*^h^*↘*_v_*_) = −2.8[−12.5,10.2]; ΔAIC(Ω_*R*:*_h_*↗*^v^*_) = −4.8[−23.4,13.6]; Fig. 4C). Given that the rational inattention model fits the data better than all other models tested here so far, these results suggest that the RL model closely approximates the solution of the optimal model (bootstrap 95% CI of ΔAIC < 0 between RL and all other models except the rational inattention model).

A recent intriguing modeling study found that, when updating the value of a chosen option, estimates must be revised more strongly following positive than negative reward prediction errors, as this guarantees that reward expectation is maximized (*44*). Crucially, this effect is amplified when decisions are corrupted by late noise. Given our finding that downstream noise has an important influence in the allocation of limited resources and consequently on decision behavior, we hypothesized that, similar to humans (*42*), learning rates in mice are also higher for correct relative to incorrect decisions in our adaptive resource allocation model. In line with these predictions, we found that learning rates were generally higher for correct relative to incorrect decisions (Δα = −249.0, bootstrap 95% CI[−465.6, −41.7]; Fig. 4B), thus suggesting that apparent irrational learning policies might lead to reward-maximizing strategies.

### Rationally inattentive neural population codes

**Fig. 5.**
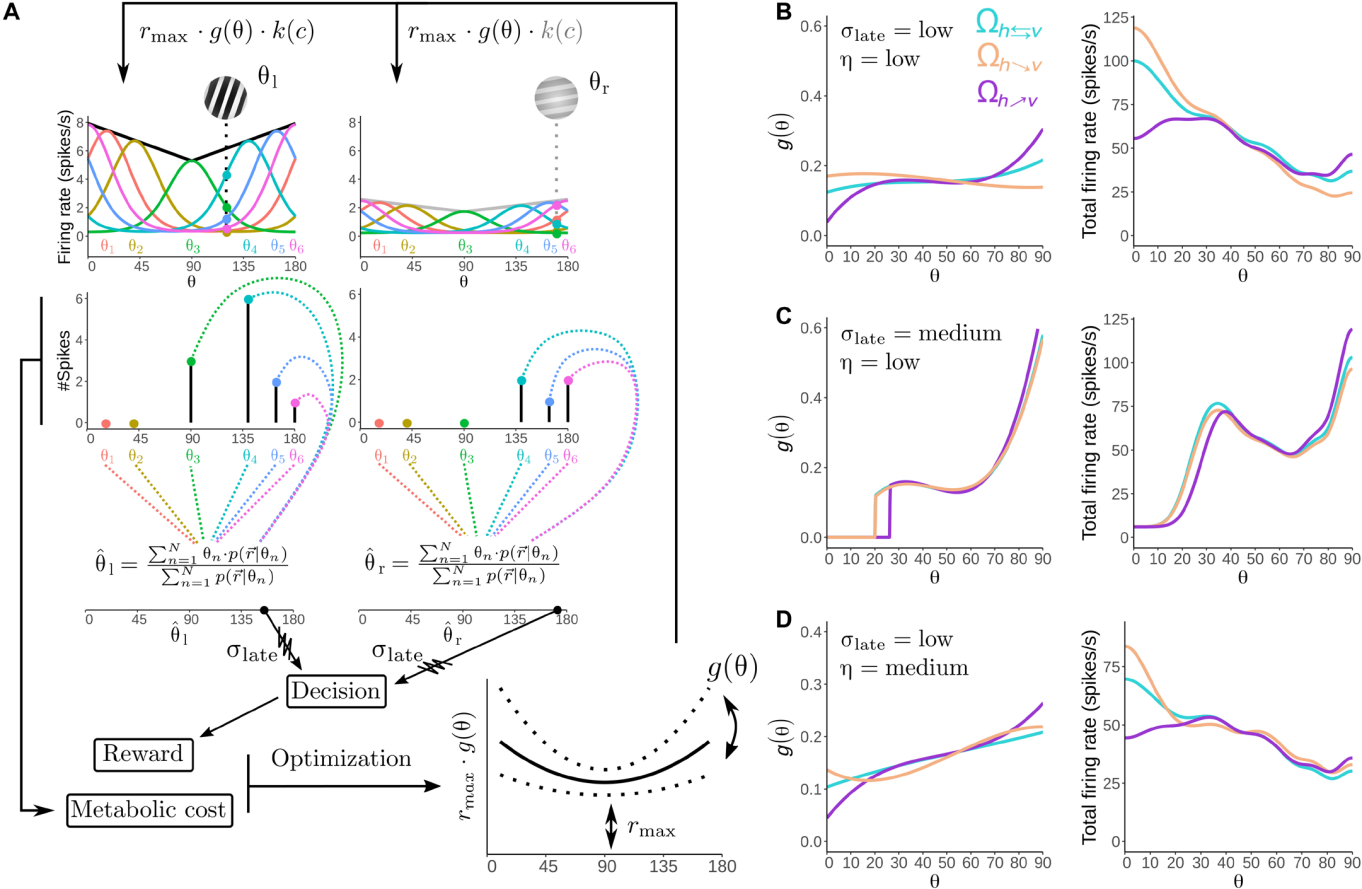
Neural network analysis. (**A**) Schematic of the neural network. Top: The preferred stimuli θ*_n_* of *N* = 30 neurons are spread through orientation space proportional to the prior distribution (six depicted). Neurons follow a bell-shaped activation pattern that is modulated by the maximum firing rate *r*_max_ and the resource gain function *g*(θ). Neural activities are, in turn, modulated by a contrast response function *k*(*c*). The two input angles θ_l_ and θ_r_ activate independent and retinotopically specific neural networks. Note that the low activation of the neurons for θ_r_ is a result of low contrast. Second row: The activation of Poisson neurons leads to a number of spikes for each neuron. Third row: BLS estimates of the input angles ${\hat{\theta }}_{l}$ and ${\hat{\theta }}_{r}$ are computed by summing over the preferred orientation of the neurons multiplied by the likelihood. Bottom: A noisy decision is made on the basis of BLS estimates of the input angles. Both the reward and the metabolic cost originating from the spikes inform the optimal *g*(θ) function and the value of *r*_max_. (**B** to **D**) Optimal solutions of *g*(θ) for different values of the metabolic cost and different levels of late noise σ_late_ for the different environments Ω (left) and the resulting total activation (as a function of input angle θ) (right). (B) Low external noise and low metabolic cost. (C) Medium external noise and low metabolic cost. (D) Low external noise and medium metabolic cost. Note that the scale of the *y* axis has changed in (D) compared to (B) and (C).

We investigated the allocation of neural resources of our sensory task in a model that is more closely related to a possible biological implementation. To this end, we implemented a neural network of V1 neurons with Poisson spiking statistics. Thus, an advantage of studying such an implementational architecture is that we can directly study the trade-off between reward intake and the metabolic costs associated to the generation of action potentials in the network (*45*). Here, we provide a brief description of the model alongside the main results (Fig. 5A; see Methods for more details). Similar to the algorithmic model, the firing rate of the neurons is determined by two multiplicative factors: (i) maximum activity *r*_max_, determining the maximum firing rate of the neurons (which is also modulated by contrast input), and (ii) *g*(θ), which determines how the firing rate gain of the neurons is distributed across the sensory space.

On the basis of this architecture, it is possible to show that the BLS estimate of the input θ can be computed by a sum over the likelihood functions based on the neural activity generated across the network $p(\vec{r}|\theta_{n})$ weighted by the preferred stimuli of the tuning curves θ*_n_*. Given that the physical prior distribution remained constant across the different environments Ω, we assume that the neural network incorporates this knowledge in its architecture, such that biologically plausible computations can be implemented during encoding and decoding operations of the network. One way to achieve this is by assuming that the locations of peak sensitivity of the neurons are spread through the orientation space proportional to the prior (*38*, *46*). Conveniently, this strategy can be incorporated by the BLS estimator via the spread of the preferred stimuli locations of the neurons. We assume that these computations occur in parallel by retinotopically specific networks that independently compute the BLS estimates of the two input angles ${\hat{\theta }}_{l}$ and ${\hat{\theta }}_{r}$. As in the algorithmic model, we allow the possibility to corrupt the estimators with downstream noise (σ_late_). The decision is associated with a reward outcome that conforms with the decision rule and the reward contingencies of the environment Ω. Crucially, we can directly relate the metabolic cost to the expected amount of spikes generated by the network. We assume the metabolic cost per spike to be equal to η. As in the algorithmic model, the goal is to find a resource gain function g* and a maximum allowed activity *r*_max_* such that the trade-off between reward and costs is optimized (see Methods and Fig. 5A).

Mirroring the results of the algorithmic model, we find that the allocation of resources in the network (measured as firing rates elicited for each input stimulus in the sensory space) depends on encoding noise, late noise, and the environmental context Ω (Fig. 5, B to D). For low levels of downstream noise σ_late_, more resources are allocated to segments of orientation space that correspond to high prior π(θ) density (Fig. 5, B and D). This intuitive result is not found when σ_late_ is increased; instead, most resources are located at those regions of orientation space that correspond to low prior density (Fig. 5C). Moreover, the neural network will spend fewer resources in the case that the cost of spiking η is high (i.e., average firing rate decreases; Fig. 5D). Thus, this model makes testable predictions for the spiking behavior of neurons in the face of prior densities, stimulus-reward association contexts, and varying levels of encoding and late noise, which can be studied in future imaging studies.

### Arousal-linked efficient regulation of behavioral variability

Past research has identified that systems regulating arousal levels, such as the locus coeruleus–norepinephrine (LC-NE) system, have a considerable impact on behavioral variability (*47*). For instance, it has been shown that large pupil baseline (i.e., pupil dilation before trial onset) is associated to slower and less accurate perceptual decisions (*48*). Moreover, in behavioral paradigms involving uncertain environments, large pupil baselines predict exploratory behavior (*49*, *50*). These findings support the idea that tonic LC-NE modes produce an enduring and largely nonspecific increase in behavioral sensitivity, which promotes flexible and exploratory control states (*50*).

**Fig. 6.**
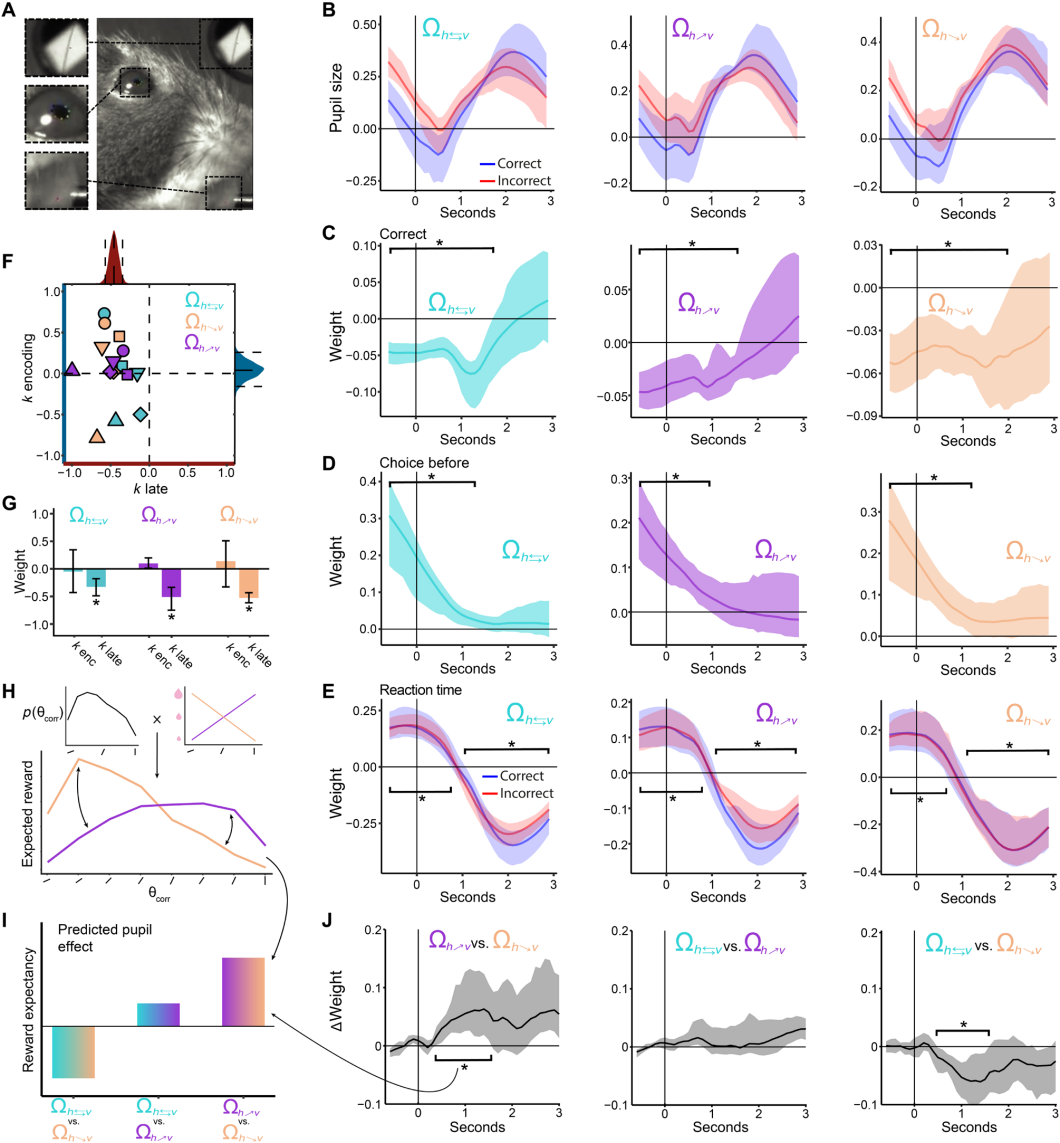
Arousal and pupil dilation analyses. (**A**) Pupil dilation and other parameters were extracted from videos during behavioral performance. (**B**) Average pupil size for each reward environment Ω for correct (blue) and incorrect (red) trials. Shaded area: 95% bootstrap CIs. (**C** to **E**) Regression coefficients are estimated for each time point on the basis of multiple linear regressions with pupil size as the dependent variable outcome. Panels show coefficient estimates for trial correctness, correctness on a trial before, and reaction time (multiple regressions ran separately on correct and incorrect trials). (**F**) Baseline pupil dilation affects downstream noise precision but not sensory precision. Regression weights of sensory precision (*k* encoding) versus downstream choice precision (*k* late) as a function of baseline pupil dilation. Individual mice are shown with different symbols and color-coded for condition. Bootstrapped distributions projected parallel to axes with mean and 95% CI are shown in dashed lines. (**G**) Bootstrapped means and 95% CIs for sensory and downstream noise parameters. (**H**) Sketch showing how expected reward values were extracted for each correct side angle. The prior distribution of angles is multiplied by the reward mapping of the respective reward environment Ω. (**I**) Sketch of the predicted reward expectancy effect on pupil size when comparing between environments. Arrows pointing to the furthest right bar illustrate the comparison between Ω_*R*:*^h^*↘*_v_*_ and Ω_*R*:*_h_*↗*^v^*_ reward environments. (**J**) Pairwise reward environment difference in weights of expected reward to phasic pupil response. All time bins marked with asterisks are significantly different from 0 (95% bootstrap CIs). These results suggest that arousal systems potentially encode expected reward distributions in a sensory-specific manner.

On the basis of this evidence, we hypothesized that, as for the case of humans, baseline pupil dilation in our task is predictive of task disengagement, that is, more incorrect and slower responses. On the basis of camera tracking, we analyzed the dynamics of pupil dilation relative to trial onset and its relation to RTs and correct responses (Fig. 6, C and E, and Methods). In line with our hypothesis, we found that higher levels of pupil baseline were associated with more incorrect and slower responses in the upcoming trial for all environments Ω (bootstrap 95 % CI < 0 for all Ω from −0.75 to 1.5 s relative to stimulus onset; Fig. 6B). Given that RTs are usually related to the degree of trial correctness (Fig. 1J), we investigated whether the effect of pupil baseline on RT was also present when splitting the data in correct and incorrect trials. We found that the effect of RT was robustly present for both correct and incorrect trials (bootstrap 95 % CI < 0 for all Ω from −0.75 to 1.5 s relative to stimulus onset; Fig. 6E). These results confirm that during high levels of tonic arousal, as in humans, mice show signatures of task disengagement. However, what mechanisms support this arousal-related behavioral variability?

Recent theories of the LC-NE system suggest that windows of high arousal might be related to adjustments in learning representations [e.g., in situations of high volatility where learning rates must be up-regulated (*51*, *52*)]. However, where does this trade-off occur during decision-making? An advantage of our rational inattention model is that it allows us to separate sensory encoding precision from stimulus-unspecific downstream noise. Therefore, we adapted our model to obtain a joint readout of change in sensory and late noise as a function of pupil baseline (parameter recovery relative to identity line *R*^2^ = 0.94). Notably, we found that, for all environments Ω and all mice, higher levels of pupil baseline resulted in a consistent reduction of downstream choice precision *k*_late_ [Δ*k*_late_(Ω_*R*:*h*⇄*v*_) = −0.33%, 95% CI[−0.48, −0.18]; Δ*k*_late_(Ω_*R*:*^h^*↘*_v_*_) = −0.55%, 95% CI[−0.63, −0.46]; Δ*k*_late_(Ω_*R*:*_h_*↗*^v^*_) = −0.52%, 95% CI[−0.78, −0.33]; individually estimated maximum likelihood estimate (MLE) 95 % CI < 0 for each mouse in each environment Ω], but not related to changes in sensory precision (Fig. 6, F and G).

Given our finding that positive prediction errors are associated with higher learning rates (Fig. 4B) and previous reports on the positive relationship between learning rates and tonic arousal (*51*), we hypothesized that following positive prediction errors, pupil baseline should be elevated. In line with this prediction (and after controlling for confounding factors; see Methods), we found that positive prediction errors were related to elevated pupil baseline of the following trial (Fig. 6D). Conversely, higher levels of phasic pupil response were related to faster RTs in the current trial (Fig. 6E). Thus, these findings suggest that arousal systems in the brain balance the costs associated to larger learning updates with lower precision in downstream circuits while preserving sensory fidelity. This supports previous suggestions of nonspecific increase in behavioral sensitivity during high tonic arousal, which, in turn, promotes flexible and exploratory control states (*49*, *50*).

### Signatures of reward distribution encoding in arousal systems

Previous work provided evidence that the arousal systems have important and computationally complex roles in rationally regulating the influence of incoming information on beliefs about dynamic and uncertain environments (*51*). However, it remains unclear whether the arousal systems signal the expectation of rewards tied to specific sensory information (which is often distributed across the whole sensory space), irrespective of the expectation of physical sensory signals. Our experimental design allows the possibility to study these arousal-linked computational mechanisms given that the distribution of sensory signals remains identical across the different environments Ω, where the only difference across environments is the learnt stimulus-reward association spanning the sensory space.

To study this possibility, we investigated phasic pupil responses as a function of sensory-specific reward expectation. First, we obtained the expected reward of each correct-side stimulus by multiplying the frequency of occurrences of these stimuli with their reward value within each reward environment Ω (Fig. 6H). If it is true that pupil responses signal the distribution of reward expectation in an adaptive manner, then we expect an interaction of the pupil responses linked to sensory-specific reward expectation across environments (Fig. 6H).

We used these values in a per-time-bin linear regression analysis on phasic pupil responses. Critically, we controlled for confounders such as trial difficulty (absolute angle difference), RT, correctness on a trial, the probability of appearance of an angle, and licking by including them as regressors. In a within-mouse, pairwise comparison, we find a significant difference between the regression weights assigned to expected rewards in different reward environments Ω as predicted by our postulated computations (Fig. 6, I and J). A positive interaction between Ω_*R*:*^h^*↘*_v_*_ and Ω_*R*:*_h_*↗*^v^*_ is expected because of the inverted angle-reward mappings between them (Fig. 6, H and I). The phasic pupil activity reveals these interaction patterns (cluster-corrected *P* < 0.05; Fig. 6J, left). The opposite pattern is predicted by the comparison between Ω_*R*:*^h^*↘*_v_*_ and Ω_*R*:*h*⇄*v*_ (Fig. 6I), which is also confirmed by the phasic pupil responses (cluster-corrected *P* < 0.05; Fig. 6J, right). On the other hand, the predicted difference between Ω_*R*:*_h_*↗*^v^*_ and Ω_*R*:*^h^*↘*_v_*_ is relatively small with a slight tendency in the positive direction (Fig. 6I). This prediction was also evident in our data (Fig. 6J, middle).

This set of results indicates that pupil size increases with rarity and size of the reward indicated for a given set of sensory stimuli in a given trial. This suggests an indirect link between arousal systems and reward expectancy, which might guide learning and redistribution of sensory resources via dopaminergic systems. A recent investigation showed that dopaminergic responses are amplified by rarity and size of reward received in a trial (*53*). In addition, they show that pupil dilation is also sensitive to these rewards after feedback, thus pointing to a fundamental interaction between noradrenergic and dopaminergic systems to guide efficient learning and decision-making behavior.

## DISCUSSION

When organisms face a decision, choices must be made on the basis of imprecise perceptions arising from the information processing limitations of the nervous system. We developed a framework to study how organisms allocate attention during sensory encoding, such that there is an advantage to being stochastic and, in some cases, myopic to incoming sensory information, because the encoding strategy guarantees that metabolic investment in more precise awareness leads to maximal reward consumption. We formulated the framework as generally as possible while conforming closely with neurobiological mechanisms so that the nature of the neural-coding constraints need not be fine-tuned independently for each particular context. In addition, we show that the information processing strategies of a rationally inattentive agent are dependent on two main factors: First, the system has limited capacity to process information not only at the early stages of sensory encoding but also at late stages of the decision-making process. Second, strategies depend on context; in our work, context was defined by the stimulus-reward associations in a given environment, but the stimulus prior remained constant at all times. We found that mice behave as rationally inattentive agents: They take into consideration their information processing limitations to develop efficient sensory encoding strategies that lead them to maximize reward consumption.

### Efficient allocation of sensory resources depends on early and late noise

Within the specific structure of our decision task, rational information processing strategies lead to results that seem unexpected and also appealingly intuitive. When information processing resources are low for both sensory encoding and downstream decision computations, the rationally inattentive agent is relatively myopic to portions of the stimulus space where the density of the prior is highest. This result goes against concepts of efficient coding, which suggest that the system should dedicate more neural resources to stimuli that occur more often (*18*, *38*, *54*, *55*). However, these suggestions usually assume that the system has a large capacity to process information and that there is no uncertainty in downstream circuits. This prediction also emerges in our task and model under the high resource-capacity assumption, but we argue that these efficient-coding assumptions hold only for cases in which organisms can process sensory information with high precision and have a perfect understanding of the task with no uncertainty about choice rules or related sources of noise in circuits dedicated to comparison, action, and learning. However, mice cannot follow human instructions and must learn task rules by trial and error, so it is reasonable to believe that behavioral variability in mice is largely affected by downstream noise (*56*). Crucially, this counterintuitive myopic behavior was evident in all the animals tested in this study, in line with the rational inattention framework. It will be left for future imaging studies to test whether higher levels of noise at both early and late stages of processing shift neural resources away from high prior density spaces.

While support for our theory from mice’s behavior is encouraging, we believe that these results might have important implications in human decision-making domains where the complexity and understanding of the task structure may be key to interpreting decision-making models. For instance, recent work has shown that a good deal of the variability in behavior of human participants in model-free and model-based learning processes might be rooted in task instructions (*57*). Thus, it is tempting to speculate that, when task understanding is poor, agents may develop apparently deviant coding strategies that might be efficient.

### Efficient allocation of sensory resources depends on stimulus-reward contingencies

A key prediction of the rational inattention framework developed here is that information processing strategies change endogenously as a function of stimulus-reward associations in a given environment despite the fact that the physical environment is held constant across all contexts. Studies of efficient adaptation typically focus on scenarios in which the statistics of the physical environment change while keeping the reward contingencies constant. However, we argue that studying scenarios in which the statistics of the physical environment remain constant is also ethologically relevant because physical environments are generally stable over longer periods of time, whereas stimulus-reward associations may change more often. We found that mice’s characteristic levels of information processing capacity, during sensory encoding and decoding, enabled them to adaptively allocate their limited resources, which appeared to be related to improving learned associations that maximized utility. This led to myopic sensory encoding in various ways depending on the contextual reward-stimulus contingencies, as predicted by the theory.

Recent developments in the neurobiology of efficient information representation suggest that neural codes should be tuned not only by stimulus frequency statistics but also by the stimuli’s impact on downstream circuits and, ultimately, on behavior (*55*, *58*). In line with this intuition, there is evidence showing that early sensory systems represent not only information about physical sensory inputs but also nonsensory information depending on the requirements of a specific task and the behavioral relevance of the stimuli (*34*, *59*). Although we did not record the activity of sensory neurons, our work provides a formal justification for these intriguing observations, where information processing resources endogenously adapt and reflect the organism’s needs. Given that noisy communication channels such as the brain always lose information during transmission, we argue that it is more efficient for the brain to adapt to the utility-maximizing rules of a particular environment at the earliest stages of sensory processing, an intuition that appears to be supported by recent imaging studies (*59*).

We note that, in our work, we constructed the prior distribution of orientations on the basis of the information coding structure in V1. However, we caution that earlier visual structures such as the superior colliculus (SC) are also essential for guiding behavior based on low-level sensory features, and it is well possible that efficient reallocation of neural resources occurs earlier than V1. The presence of orientation-selective neurons in the SC is well known in rodents (*60*–*64*), and this structure appears to play a causal role in visual perception tasks involving orientation judgments (*65*). Previous work studying the distribution of orientation tuning in the SC shows that not all orientations are represented equally for all visual field locations. One such study showed that parts of the SC located at intermediate elevations tend to prefer horizontal orientations (*61*). However, the reason why we opted to define the prior distribution based on the study of Stringer *et al.* (*27*) is that the investigators recorded and characterized the structure of V1 orientation tuning based on ∼20,000 neurons, which is about two orders of magnitude more neurons than the studies targeting the SC, thus providing us with higher-quality information about the distribution of orientation coding in V1. However, future imaging studies will be necessary to determine at which information processing stages efficient reallocation of sensory information occurs such that goal-oriented behavior is optimal according to our theory.

### A biologically inspired bounded-rationality RL algorithm

In this work, we studied the possibility that an RL algorithm supported by operations that appear neurologically plausible could provide insights into how information processing resources could be adaptively and optimally allocated through trial-by-trial experience. We leveraged recent discoveries suggesting that the brain can represent and update information about rewards in a distributional manner (*40*) and that functional remapping of task-relevant early sensory areas can be achieved with top-down feedback from decision-relevant circuits that encode prediction error signals and task rules (*59*). Recent work revealed that dopamine neuron ensembles generate activity patterns that signal sensory-specific prediction errors (*66*), thus providing further support for our algorithmic architecture. Last, adjustment of sensory gain is achieved via divisive normalization, an operation that has been related to the implementation of optimal attentional reallocation (*43*). We found that an algorithmic architecture of this kind allocates sensory resources in a context-dependent manner, similar to the patterns predicted by our rational inattention theory. Although, in this model, sensory resources are allocated dynamically and thus endogenously through trial-to-trial experience, in our current RL specification, the parameters determining the learning rates and the maximum allowable sensory precision are not endogenously estimated but fitted to the data. A similar problem emerges with the original specification of distributional RL (*40*): The agent needs to find a probability distribution consistent with the set of optimal updating operations, which requires a precise coordination in every time step of all neurons involved in RL computations. Although recent computational formulations provide hints on how this problem could be tackled (*67*, *68*), it remains unclear how to connect these distributional RL computations to a biologically plausible algorithm that applies to arbitrary stimulus-reward association contexts such that reward expectation is maximized. This topic deserves attention and should be studied further.

### Arousal systems balance learning costs and carry reward distribution information of sensory signals

Our computational model allowed us to investigate the role of adaptive regulation of arousal systems on sensory and nonsensory downstream imprecision. This approach revealed that nonspecific increase in behavioral sensitivity is associated with high levels of tonic arousal, but not directly related to sensory sensitivity. These results indicate that arousal systems may balance the costs associated with larger learning updates, which lead to high tonic arousal states and lower precision in downstream circuits while preserving sensory fidelity. This mechanism may have the benefit of leaving intact learning updates in sensory systems as a function of recently experienced sensory stimuli. Together, these results provide further support for the mechanisms of arousal-mediated adaptive gain control theory (*69*), indicating that some aspects of inattentive behavior might be rational because the brain must operate under limited capacity constraints.

In addition, we found that arousal systems carry reward expectancy information about specific segments of the sensory space. An important implication of such neurocomputational signature could be that agents track the utility distribution and volatility of the environment so that they can more accurately modulate prediction error signals. Recent work has shown that dopaminergic responses are amplified not only by the rarity of a reward received in a trial but also by its size (*53*). Thus, bottom-up noradrenergic reward expectation signals might be combined with top-down dopaminergic learning signals to efficiently guide reallocation of attentional resources in sensory systems. Thus, our computational modeling approach and behavioral paradigm implemented in rodents open the way to detailed investigations of the mechanisms underlying tight and essential interactions between arousal and dopaminergic systems. For instance, it will be interesting to investigate in future studies whether manipulations of neural systems regulating arousal (e.g., the LC, but there are also other key players) modulate processing of early or downstream information processing during behavior.

### Neurobiological predictions

Our finding that rational inattention theories can be studied in laboratory rodents, which allow tractable access to defined neural circuits, enables the formulation and future study of specific neurobiological predictions that emerge from our theory and related behavioral results. Here, we list some of these neurobiological predictions that might help confirm or falsify the theory.

One key prediction of our model is that neural responses to sensory signals should preferentially increase for stimuli associated with more reward, even when the relative prevalence of these signals in a given environment (i.e., the sensory prior) remains constant across time. Specifically, for visual tasks such as the one described here, we predict that, during reward-based learning, stimuli-reward associations across the sensory space (e.g., of oriented edges) would progressively recruit a greater number of neurons, and/or higher firing rates, relative to stimuli that are less rewarded, crucially, while also considering the relative frequency at which each stimulus in encountered. Furthermore, on grounds of efficient resource use, which is at the heart of our model, we speculate that such selective reallocation of neural resources to stimuli associated with higher rewards (rather than simply most common stimuli) might be seen already at the lowest levels of the visual system such as the retina. Such reward-dependent sensory filtering would be efficient in the sense that it will prevent “low-benefit” sensory information from demanding neural resources in downstream circuits. The biological feasibility of such early filtering and the experimental tools for probing this further have been recently described for the retina of awake behaving rodents (*70*).

A related neurobiological prediction is that the stimulus-response remapping in neural populations, and/or individual neurons or synapses, is mediated by reward-engaged neuromodulatory systems. We speculate that dopamine and/or noradrenaline inputs to early sensory systems may be candidates for these modulations because they are engaged by reward experience and are also linked to control of neuronal sensory gain (*69*, *71*, *72*). Our specific experimental prediction is that temporally targeted silencing of reward-associated dopaminergic or noradrenergic neurons (e.g., optogenetic silencing of LC-noradrenaline of VTA-dopamine neurons or of their specific axonal projections to sensory or decision-related circuits) would suppress the reward-based phasic pupil responses described by our model, in parallel with suppressing reward-based remapping in early sensory structures.

In addition, in future studies, it will be important to directly quantify efficient metabolic regulation given (i) information processing costs and (ii) the scarcity of neural resources, thus allowing us to test the hypothesis that the brain evolved energy-efficient coding strategies that maximize information transmission per unit energy, for instance, as measured via adenosine triphosphate (ATP). Our theory and behavioral paradigm predict that, in environments with high sensory noise (i.e., low visual contrast environments), it is not worth the effort of investing metabolic resources to promote discrimination performance (e.g., see figs. S6 and S7). We predict that, for visual sensory systems, a way to implement this solution might be to generally broaden the tuning curves of neurons responsive to oriented edges across the sensory space. The reason for postulating this quantifiable prediction is that adaptive broadening of orientation tuning is related to AMPA receptor conductance decrease, which, in turn, reduces synaptic ATP use, and should directly affect sensory discrimination performance (*73*). Experiments of this sort should help establish the link and quantify the balance between efficiency in adaptive information processing and metabolic consumption costs, which is crucial to promote reward maximization of the organism via goal-directed behavior.

### Broader impact

Rodents have become an important model system in the study of decision behavior (*74*). Here, we show that these organisms might be key to gaining a deeper understanding of the neurobiology underlying decision-making theories, which currently have a large impact in other disciplines such as medicine and economics. For instance, processes derived from rational inattention theories appear to be essential in guiding policy-making in both microeconomic and macroeconomic settings (*75*). In other fields, recent investigations have developed theories that attempt to provide normative accounts of complex neuropsychiatric conditions such as autism and schizophrenia, which have been characterized by deficits in performing optimal inference (*76*). However, these theories generally ignore the normative foundation that organisms must optimize behavioral processes in light of biological restrictions on information processing (*77*). Thus, the growing battery of molecular and imaging tools that is becoming available for use in rodents will enable a deeper understanding of the neurobiological underpinnings of limited cognition and apparently irrational decision behavior. Thus, the corroboration of our theory using mice as a model organism opens the door to new directions that might be instrumental in the refinement and translation of these theories to applied settings in medicine, economics, and related social sciences.

## METHODS

### Animal subjects

All animal experiments were performed in accordance with the Animal Welfare Ordinance (TSchV 455.1) of the Swiss Federal Food Safety and Veterinary Office and approved by the Zurich Cantonal Veterinary Office. Subjects were adult (at least 8 weeks old) C57BL/6 mice (six males and one female). Mice were kept on a reversed 12-hour light/12-hour dark cycle, and all experiments were performed during the dark phase.

### Surgery

To ensure that the head was held at a natural angle in the behavioral apparatus, a custom-printed headplate (Protolabs Inc.) was fitted to the cranium of the animal using dental cement. For implantation of headplates, mice were anesthetized with isoflurane (1 to 2%) and their body temperature was kept constant with a heating pad. To protect the eyes from drying out, artificial tears were applied several times during the surgery. Animals’ heads were held in fixed stereotaxic coordinates using a stereotaxic frame (Kopf Instruments). Lidocaine was applied to the skin on the cranium before it was incised to gain access to the cranium. The headplate was centered over the bregma and fixed with dental cement. The skin was glued to the side of the cement to close the wound. The animal was kept on analgetics for 3 days after surgery and monitored for another 2 days past that. The mice were allowed to recover from the surgery for at least 10 days before starting experiments.

### Behavioral setup and tasks

The specifics of the behavioral setup, as well as most of the parts used to construct it, are very similar to those developed in previous work (*78*) and widely adopted by the International Brain Laboratory (*79*). Briefly, the animal is head-fixed on a platform in front of a screen, with a small wheel that the animal can turn left or right using its front paws. Sound signals were delivered through a speaker placed just under the screen, in front and slightly below the animal. A small drinking spout was placed in front of the animal’s mouth for delivery of liquid reward (strawberry milkshake). The head-fixed mice were trained to look at two gratings presented on the screen and pick the one that is more vertically oriented by turning the wheel with their forepaws, which was coupled to the position of the two stimuli (Fig. 2B).

Following recovery from surgery, the animals were put on a food restriction schedule (not dropping below 85% of initial weight). At the end of each training session, the animals were weighted and additional amount of food was provided, dependent on the percentage of initial weight. For 3 days, the animals were habituated to being head-fixed and handled by the experimenter, with milkshake being delivered in random intervals during increasing periods of head fixation. Then, mice were trained on the initial task, in which only a single stimulus appeared on either left or right side of the screen, which they had to move to the center of the screen (response location), to obtain a milkshake reward. After they mastered the simple selection task, the second grating was introduced (more horizontal/distractor), initially at low contrast but progressively increasing contrast across sessions as the animal learned. After the second grating was completely introduced (contrast, 1) and session performance was above 60%, we started varying the contrasts of the two gratings independently across three levels (0.3, 0.6, and 1). Contrast was defined as the proportion of change from the middle color range (gray) on the screen to either the lightest (white) or darkest (black). For example, contrast difference of 0.3 meant that the brightest stripes in the grating were 30% of the way from gray to white and darkest stripes were 30% of the way from gray to black. During the entire training procedure, we kept the orientation stimuli θ that were drawn from the prior distribution π(θ) (see Fig. 1A). Once the animals reached good performance on the task (>60% across difficulties), we introduced different mappings of reward to orientation (Ω). In each of these mappings, the reward size obtained for correctly picking the more vertical orientation was dependent on that orientation. We then ran each animal in each of the reward environments for at least 20 sessions. Here, we studied adaptation to a particular environment Ω after seven sessions of experience. Considering longer adaptation periods does not affect the main conclusions of our work.

To keep the volumes of milkshake constant, the opening times of click valves (NResearch, 161K011) were calibrated, before each day of training, to deliver a constant volume of milkshake. This was achieved by opening the valves repeatedly for different durations and subsequently fitting a curve, describing the relation between opening time and milliliters of delivered milkshake. Milkshake amounts in microliters were mapped to the correct-side angle, dependent on the reward mapping environment Ω. In the Ω_*R*:*_h_*↗*^v^*_ environment, the reward mapping was a linear increase from horizontal (0°, 1 μl) to vertical (90°, 8 μl). The reward mapping for the Ω_*R*:*^h^*↘*_v_*_ environments was exactly the opposite, with horizontal grating yielding 8 μl and vertical yielding 1 μl. In the Ω_*R*:*h*⇄*v*_ environment, all angles yielded 5 μl. The illustration of the reward mappings for each environment Ω is shown in Fig. 1E, and the exact amounts are shown in table S1.

The stimuli were Gabor patches each spanning 15 visual degrees and with the phase of the grating randomized between trials. Decision was considered made when one of the gratings was brought to the middle of the screen. Correct choice yielded a drop of strawberry milkshake, and incorrect led to white noise being played for 0.5 s and a 2-s timeout before next trial (in addition to the inter-trial interval (ITI) of 1.5 to 2.5 s). To counteract bias, when animals might be content with just turning the wheel one way and getting 50% of rewards, incorrect trials were repeated (the correct side was kept the same, but orientations were resampled), but data from these trials were not used in the final analysis. To check that this did not influence the results, two animals were also successfully trained without repeating incorrect trials. For all experimental conditions, the orientations of the two gratings were picked from a manually set prior distribution of orientations (Fig. 1A), with the difference between the two ranging from 20° to 90°, in increments of 10°. In a small percentage of trials, the two orientations were the same, in which case, a random side choice delivered the reward. For half a second after stimulus presentation, the stimulus position was not coupled to wheel rotation, but the animal was not punished for turning it at this time (Fig. 1C). Signal tone (12 kHz), played for 0.2 s, cued the end of wheel-uncoupled phase, and the animal could make the response.

As a reward, strawberry milkshake was used. It contains partially skimmed milk, 8% concentrated strawberry juice, sugar, pasteurized whole egg, dextrose (2 g/100 g), maltodextrin (2 g/100 g), soluble fiber (inulin), stabilizer E339, concentrated beetroot juice, flavors, concentrated milk mineral, carrageenan thickener, and vitamins E, B6, B2, B1, and D.

### Pupil size analysis

To track the pupil and licking, we filmed the animal from the side, illuminated by infrared light, using a FLIR Blackfly camera. Pupil edges and licking responses were extracted using DeepLabCut (*80*), and eye diameter was calculated from the obtained videos. Trials were synced to video using a small white square in the corner of the screen that appeared at stimulus onset. The area of the pupil was calculated from the points tracked by DeepLabCut, and the trace for each daily session was low-pass–filtered with a cutoff frequency of 20 Hz to remove noise. Pupil size was then *z*-scored within session. Pupil size was binned into 0.2-s bins, with the binning window moving by 0.1 s, further smoothing the data. All multiple linear regressions studied here were run on binned time points, with pupil size as a dependent variable. Factors in the regression included the following: absolute angle difference, contrast sum, RT, correct side orientation, correctness on a trial, correctness on the previous trial, and binned licking. Furthermore, we split the data into correct and incorrect trials and ran the same regression but excluded the trial correctness parameter. We also measured phasic pupil responses by subtracting the baseline 0.5 s before stimulus onset from each trial pupil size. To investigate the arousal responses based on reward expectancy, we ran a bin-time regression to predict the phasic pupil response, same as for the nonbaseline corrected data.

### Descriptive behavioral models

Here, we studied six DMs, each with different levels of complexity, which allowed us to evaluate basic behavioral signatures in all three different reward mapping conditions. This family of models does not explicitly use information about the statistics of the environment and condition-dependent behavioral goals.

On each trial, mice face two alternatives l and r with corresponding orientations θ_l_ and θ_r_, which are shown on the left and right side of the screen, respectively (Fig. 1B). The goal of the mice in all three stimulus-reward environments Ω is to select the alternative that is closer to 90°. In the description of all models (unless otherwise specified), we assume that the orientations are mapped to an abstract “verticality” space from 0° to 90° (e.g., θ = 170° is mapped to θ = 10°). In DM1, the probability of choosing alternative l in trial *n* is given by

$$P_{n}(\text{choose}\;l|\theta_{l},\theta_{r})=\Phi (\frac{\theta_{l}-\theta_{r}}{\sigma \sqrt{2}}+\beta_{0}+\beta_{1}D_{n-1})(1-\lambda )+\frac{\lambda }{2}$$ (3)

where Φ() is the normal cumulative density function, σ represents the degree of sensory noise in the representation of θ (which in DM1 model is fixed for all orientations), β_0_ captures potential side biases, β_1_ captures biases caused by the decision *D* in the last trial (*n* − 1), and λ captures potential lapse rates (to simplify notation; in the remainder of the model descriptions, we will drop the trial indexes *n*).

In DM2, we studied the possibility that different levels of contrast are associated to different levels of sensory noise. Therefore, DM2 is given by

$$\begin{array}{c} P(\text{choose}\;l|\theta_{l},\theta_{r})=\\ \Phi (\frac{\theta_{l}-\theta_{r}}{\sqrt{\sigma {\left(c_{l}\right)}^{2}+\sigma {\left(c_{r}\right)}^{2}}}+\beta_{0}+\beta_{1}D)(1-\lambda )+\frac{\lambda }{2} \end{array}$$ (4)

where, in this case, the noise level of each alternative is a function of contrast *c*. In this model, we fit three different noise levels for the different levels of contrast used in this study.

In DM3, we investigated the possibility of “risk seeking/aversion” depending on the level of sensory uncertainty of each alternative induced by the different levels of contrast

$$\begin{array}{c} P(\text{choose}\;l|\theta_{l},\theta_{r})=\\ \Phi (\frac{\theta_{l}-\theta_{r}}{\sqrt{\sigma {\left(c_{l}\right)}^{2}+\sigma {\left(c_{r}\right)}^{2}}}+\beta_{0}+\beta_{1}D+\beta_{2}(c_{l}-c_{r}))(1-\lambda )+\frac{\lambda }{2} \end{array}$$ (5)

where β_2_ determines the strength of risk seeking/aversion (with negative values corresponding to apparent risk aversion). DM4 is identical to DM3 with the exception that DM4 allowed separate lapse rates for the left and right choices.

DM5 is similar to DM3, with the difference that it includes a parameter (β_3_) coding for reward size that interacts with the chosen side in trial *n* − 1

$$\begin{array}{c} P(\text{choose}\;l|\theta_{l},\theta_{r})=\Phi (\frac{\theta_{l}-\theta_{r}}{\sqrt{\sigma {\left(c_{l}\right)}^{2}+\sigma {\left(c_{r}\right)}^{2}}}+\beta_{0}+\beta_{1}D+\\ \beta_{2}(c_{l}-c_{r})+\beta_{3}{\left(D*R\right)}_{n-1})(1-\lambda )+\frac{\lambda }{2} \end{array}$$ (6)

where *D* is the decision made in trial (*n* − 1) and *R* is the amount of reward received in trial *n* − 1. Last, DM6 incorporates all the parameters included in models DM4 and DM5.

### Rational inattention model

We studied the possibility that mice behave according to the rules of optimal statistical inference in estimating the orientation of each choice alternative. Crucially, here, we take into consideration the fact that organisms have limited capacity to process information, and therefore, neural systems must allocate these limited resources according to each reward mapping context to maximize the amount of reward consumed over the course of many trials and days.

We assumed that, for a given input θ_0_, the mouse makes an internal measurement *m*(θ_0_) that is corrupted by sensory noise. Crucially, the level of sensory noise will depend on how many resources the system dedicates to the measured orientation (described in detail below). Each time orientation θ_0_ is presented to the observer; it results in different measurements *m_i_* described with a conditional probability function *p*(*m_i_*∣θ). On each trial *i*, the observer computes a posterior distribution *p*(θ∣*m_i_*) by combining the physical environmental prior distribution π(θ) with the likelihood of the measurement *p*(*m_i_*∣θ). Then, the mouse applies a decoding rule to obtain a posterior estimate $\hat{\theta }(m(\theta ))$. Here, we assume that mice compute the expected value of the posterior distribution: *E*[*p*(θ∣*m*)]. We assume that, on each trial, the mouse independently estimates ${\hat{\theta }}_{l}$ and ${\hat{\theta }}_{r}$ for the input orientations θ_l_ and θ_r_, respectively.

The measurement *m* follows a von Mises distribution

$$p(m|\theta )\propto e^{k(c)g(\theta )\text{cos}(\theta -m)}$$ (7)

where the precision of the measurement is determined by two multiplicative factors: (i) *k*(*c*), which is a function of the contrast level for a given orientation in a particular trial (see Eq. 8), and (ii) *g*(θ), a resource gain function. Here, *g* acts as a control mechanism that regulates resource allocation in the orientation sensory space.

Activity of visual neurons as a function of contrast has been well described by the following function

$$k(c)=\frac{k_{\text{max}}c^{q}}{c^{q}+c_{50}^{q}}$$ (8)

where *k*_max_ is the maximum activity of the neurons and *q* and *c*_50_ specify the slope and semisaturation point of the contrast response function (*81*). Given that the variability of cortical neurons approximates a Poisson distribution, one can assume that the relative variability in the measurement σ(*c*) decreases in inverse proportion to the square root of the cortical activity. Because the precision in the von Mises distribution can be defined as *k* = 1/σ^2^, neural precision as a function of contrast can be described by Eq. 8.

If we constrain *g*(θ) > 0 for all θ and to be a normalized function (we formally explain how *g* is estimated below) and assume a cost function *K* that provides information about the resources used to encode visual information, one can formulate an optimization problem to find the optimal allocation of resources in the orientation space for (i) a given physical environmental prior π(θ), (ii) a given contrast response function *k*(*c*), (iii) reward outcomes associated to decision-outcome rules in a given context or environment Ω, and (iv) downstream noise σ_late_ that is not related to sensory encoding. Formally, the goal is to find a resource gain function g* and the maximum allowed activity *k*_max_ (with an underlying contrast response function; Eq. 8) such that (Eq. 2)

$$\underset{g,k_{\text{max}}}{\text{max}}\;E[\text{reward}|\Omega ,k(c),\sigma_{\text{late}}]-K(\bar{k},\eta )$$

where *c* is the set of contrasts that the animal experiences. We model the sensory precision cost *K* as a linear function of the average precision $\bar{k}$ that the agent invests on solving the inference problem, thus $K(\bar{k},\eta )\equiv \eta *\bar{k}$, where η > 0 indicates how much the cost scales with average precision $\bar{k}$ defined as

$$\bar{k}=\int_{c}\int_{\theta }\pi (\theta )g(\theta )k(c)\;d\theta \;\mathrm{dc}$$ (9)

We note that we have not included costs associated to downstream precision 1/σ_late_. However, if we assume that σ_late_ influences choices (and therefore reward expectation in Eq. 2), including such cost in *K* as an additive, then factor does affect the estimation of the optimal allocation of resource gain *g*.

Here, we do not use information transmission as optimization criteria as classically assumed in the rational inattention literature, which is defined as follows

K=ηI(θ;m) (10)

where *I*(θ; *m*) denotes the mutual information, which measures the expected reduction in uncertainty after observing a signal *m*

I(θ;m)=H(θ)−E[H(θ∣m)] (11)

where *H*(θ) denotes the entropy of the prior distribution π(θ) and the second term measures the expected reduction in uncertainty after observing the signal. While this cost function has several benefits (such as mathematical tractability for relatively simple problems), it also has been pointed out that specifying attention costs to be linear in entropy reduction might be problematic, in particular, when applied to problems of sensory perception (*25*). In any event, for the case of sensory perception and in our experimental paradigm, we argue that it is more appropriate to assign costs to the amount of energy invested in generating neural activity as defined above. Nevertheless, we show that the two cost functions generate similar qualitative predictions in the resource allocation function *g* (fig. S4) and also study the relationship between expected precision and expected uncertainty reduction (fig. S5).

The next step is to specify the optimization problem. For instance, in the “constant reward for correct decisions” condition (i.e., environment Ω_*R*:*h*⇄*v*_), the agent aims at minimizing the expected probability of errors plus its associated costs

$$\frac{1}{\mathrm{IJ}}\sum_{i=c_{l}}^{I}\sum_{j=c_{r}}^{J}\;\iint_{\theta }P(\text{error}|\theta_{l,i},\theta_{r,j})\pi (\theta_{l})\pi (\theta_{r})\;d\theta_{l}d\theta_{r}+K(\bar{k},\eta )$$ (12)

where indexes *i* and *j* reflect the different levels of contrast (three in our experiments) applied to the left and right orientation inputs, respectively. Here, we still need to define the probability of making an erroneous response *P*(error). In our observer model, a source of decision errors is caused by variability in the estimates $\hat{\theta }(m)$ due to measurement errors *m*, thus resulting in a conditional probability of estimated orientation given the true stimulus orientation $p(\hat{\theta }(m)|\theta_{0})$ (to simplify notation, we leave occasionally the dependence on *m*). Here, we assume that the distribution of estimators $p(\hat{\theta }(m)|\theta_{0})$ is Gaussian, and therefore, it will be convenient to compute its expected value $E[\hat{\theta }(m)|\theta_{0}]$ and variance $\text{Var}[\hat{\theta }(m)|\theta_{0}]$, which are given by

$$E[\hat{\theta }(m)|\theta_{0}]=\text{atan}2(a,b)$$ (13)

and

$$\text{Var}[\hat{\theta }(m)|\theta_{0}]=-2\text{log}\;(\sqrt{a^{2}+b^{2}})$$ (14)

respectively, with

$$a\equiv \int \text{sin}\;(\hat{\theta }(m))p(m|\theta_{0})\mathrm{dm}$$ (15)

and

$$b\equiv \int \text{cos}\;(\hat{\theta }(m))p(m|\theta_{0})\mathrm{dm}$$ (16)

As in the DMs, here, we assume that the orientation estimations are mapped to a verticality space, and therefore, the probability that the mouse selects θ_l_ is given by

$$P(\text{choose}\;l|\theta_{l},\theta_{r})=\Phi (\frac{E[{\hat{\theta }}_{l}|\theta_{l}]-E[{\hat{\theta }}_{r}|\theta_{r}]}{\sqrt{\text{Var}[{\hat{\theta }}_{l}|\theta_{l}]+\text{Var}[{\hat{\theta }}_{r}|\theta_{r}]}})$$ (17)

In addition to sensory precision in the coding of orientation *k*(*c*)*g*(θ) (see Eq. 1), we also account for late noise in the decision stage (that is, post-decoding noise), which may capture any unspecific forms of downstream noise occurring during the response process that are unrelated to the estimation of orientation per se. We assume this late noise to be unbiased and Gaussian distributed $N(0,\sigma_{\text{late}}^{2})$; therefore, it can be easily added to our model as follows

$$P(\text{choose}\;l|\theta_{l},\theta_{r})=\Phi (\frac{E[{\hat{\theta }}_{l}|\theta_{l}]-E[{\hat{\theta }}_{r}|\theta_{r}]}{\sqrt{\text{Var}[{\hat{\theta }}_{l}|\theta_{l}]+\text{Var}[{\hat{\theta }}_{r}|\theta_{r}]+\sigma_{\text{late}}^{2}}})$$ (18)

Hence, the probability of an erroneous decision *P*(error) in Eq. 12 can be defined as follows

$$P(\text{error}|\theta_{l},\theta_{r})=\Phi (\frac{-|E[{\hat{\theta }}_{l}|\theta_{l}]-E[{\hat{\theta }}_{r}|\theta_{r}]|}{\sqrt{\text{Var}[{\hat{\theta }}_{l}|\theta_{l}]+\text{Var}[{\hat{\theta }}_{r}|\theta_{r}]+\sigma_{\text{late}}^{2}}})$$ (19)

With these definitions, we can formulate the optimization problem for the remaining reward mapping conditions. For the environment Ω in which the amount of received reward *R* for correct responses is linearly mapped to the degree of verticality, where horizontal (*h*) orientations are mapped to the smallest reward and vertical (*v*) orientations are mapped to the highest reward (environment denoted Ω_*R*:*_h_*↗*^v^*_), and no reward is received for incorrect responses, the goal is to find the allocation of resources that minimizes the following expression

$$\begin{array}{c} \frac{1}{\mathrm{IJ}}\sum_{i=c_{l}}^{I}\sum_{j=c_{r}}^{J}\;\iint_{\theta }\text{max}\;(R(\theta_{l}),R(\theta_{r}))\times \\ P(\text{error}|\theta_{l,i},\theta_{r,j})\pi (\theta_{l})\pi (\theta_{r})\;d\theta_{l}d\theta_{r}+K(\bar{k},\eta ) \end{array}$$ (20)

For the environment Ω in which the amount of received reward *R* for correct responses is linearly mapped to the degree of verticality, where horizontal (*h*) orientations are mapped to the highest reward and vertical (*v*) orientations are mapped to the smallest reward (environment is denoted as Ω_*R*:*^h^*↘*_v_*_), and no reward is received for incorrect responses, the goal is to find the resource gain function *g*(θ) and *k*_max_ that minimizes the following expression

$$\begin{array}{c} \frac{1}{\mathrm{IJ}}\sum_{i=c_{l}}^{I}\sum_{j=c_{r}}^{J}\;\iint_{\theta }\text{min}\;(R(\theta_{l}),R(\theta_{r}))\times \\ P(\text{error}|\theta_{l,i},\theta_{r,j})\pi (\theta_{l})\pi (\theta_{r})\;d\theta_{l}d\theta_{r}+K(\bar{k},\eta ) \end{array}$$ (21)

Recall that, in all cases, the decision rule is to choose the orientation that is more vertical. Therefore, in the Ω_*R*:*^h^*↘*_v_*_ environment, we are actually asking the mice to choose the orientation that is mapped to the smallest reward; however, in this environment, choosing the smallest orientation on any given trial is actually the decision that guarantees reward receipt.

The next step is to describe a methodology that allowed us to find potential solutions in our resource allocation problem. Our approach is to estimate the minimum achievable reward loss (plus costs) in a given environment Ω by finding the minimum achievable value over a flexible parametric family of possible functions *g*(θ), with the following properties

$$\int g(\theta )\;d\theta =1,\;\frac{\mathrm{dG}}{d\theta }>0$$ (22)

Here, we assume a finite-order polynomial function *G*(θ) consistent with the property that *g*(θ) = *G*(θ)′. This requires *G*(0) = 0 and *G*(1) = 1, and therefore, *G*(θ) can be written in the form

$$G(\theta )=\theta [1+(\theta -1)(g_{0}+g_{1}\theta +\ldots +g_{p}\theta^{p})]$$ (23)

with the properties described in Eq. 22, where *g_p_* = {*g*_0_, …, *g_p_*} is a set of parameters over which we optimize. Note that, for a large enough value of *p*, any smooth function can be well approximated by a member of this family. Also note that, for the case *g* = 0, …, *g_p_* = 0, the resource allocation problem is given by a rule that assigns equal amount of resources to all orientations θ.

In this study, we use a parameter vector *g_p_* of order *p* = 2 (we found that using a higher order did not significantly improve the optimal solutions). Given the symmetry of the prior distribution relative to θ = π, we estimate an initial $\tilde{g}(\theta )$ for the space θ ∈ [0, π] and then defined $g(\theta )=\tilde{g}(\theta )⊕\text{flip}(\tilde{g}(\theta ))$, where the operator ⊕ denotes concatenation and the operator flip() denotes vector reversal.

### Rational inattention model predictions

We used the empirical distribution of the input stimuli and the distribution of rewards (i.e., drops of milkshake) used in our experiments to derive predictions of the resource gain function *g*(θ) and the maximum activity allowed *k*_max_, which minimized reward loss (plus its associated sensory precision cost *K*). Given that we were interested in studying how resource allocation is potentially influenced by different levels of downstream noise σ_late_, we found the optimal parameter vector *g_p_* and *k*_max_ for different combinations of η and σ_late_ (Fig. 2 and figs. S3 and S4).

MDS was computed using standard techniques (*82*) by first generating a matrix of pairwise “dissimilarities” between each angle input in steps of 10°. In our case, dissimilarities correspond to psychometric distances defined as the probability of selecting the correct orientation. To guarantee that distances were 0 for the diagonal matrix elements, as it is generally required in MDS algorithms, we subtracted 0.5 from all elements of the matrix. Given that generating and visualizing MDS maps benefit from smooth dissimilarity matrices, we pooled the data across all mice for a given environment.

### Applying the rational inattention model to empirical data

To fit the inference model to the empirical data in a way that was comparable to the full DM (DM3; see above) we used

$$P(\text{choose}\;l|\theta_{l},\theta_{r})=\Phi (z)$$ (24)

with

$$z=\frac{E[{\hat{\theta }}_{l}|\theta_{l}]-E[{\hat{\theta }}_{r}|\theta_{r}]}{\sqrt{\text{Var}[{\hat{\theta }}_{l}|\theta_{l}]+\text{Var}[{\hat{\theta }}_{r}|\theta_{r}]+\sigma_{\text{late}}^{2}}}+\beta_{0}+\beta_{1}D+\beta_{2}(c_{l}-c_{r})$$ (25)

For all models, we are interested in finding the set of parameters β, *c^q^*, $c_{50}^{q}$, and σ_late_. For the endogenous model, we were, in addition, interested in finding η. For the exogenous model, we also found *g* and *k*_max_. Notably, the endogenous model has the same number of parameters as DM3. In addition, note that we dropped the lapse rate parameter λ from the rational inattention model. We reasoned that the rational inattention model rationalizes the apparent lapse rates that emerge in the DMs. Including λ as a parameter in our model led to an estimated valued λ ≈ 0 for all mice, thus confirming our intuition. To study whether the reallocation of limited resources is necessary to explain the data, we also considered a model where *g* = {0,0,0}, which corresponds to a model where mice allocate their resources equally across the whole sensory space.

### Learning model

We investigated whether an RL model that incorporates the same coding constraints of the ideal observer model would generate similar performance. That is, in the learning model, we do not directly find the parameters *g* of the resource gain function *g*(θ), but this function is continuously updated on the basis of trial-to-trial experience via RL (Fig. 4A).

Assume that, in any given trial at time *t*, a mouse chooses alternative $\tilde{\theta }$. After this decision, the mouse receives reward *R_t_* that depends on the chosen option $\tilde{\theta }$ according to decision-outcome rules of environment Ω (Fig. 1E). In addition, here, we assume that mice evaluate their degree of confidence *C_t_* on the basis of the decision in trial *t*. Here, we define confidence on the basis of the statistical definition of confidence, that is, the probability that the chosen option is correct given the chosen option: $C_{t}=p(\text{correct}|\tilde{\theta })$. Information about *R_t_* and *C_t_* is then used to update a reward distribution vector $\hat{R}(\theta )$ (mapping orientation stimuli to reward) using the RL rule

$${\hat{R}}_{t}={\hat{R}}_{t-1}+\alpha_{t}\cdot \delta_{t}$$ (26)

where α is defined as the learning rate, which can take one of two values that depend on positive and negative prediction errors

$$\alpha_{t}=\{\begin{array}{cc} C_{t}/(C_{t}+{\tilde{\alpha }}^{-}) & \text{if}\;\delta_{t}\leq 0\\ C_{t}/(C_{t}+{\tilde{\alpha }}^{+}) & \text{if}\;\delta_{t}>0 \end{array}$$ (27)

with $\tilde{\alpha }\geq 0$. This implies that, if $\tilde{\alpha }\to 0$, then the learning rate α → 1 and confidence *C_t_* have little influence. On the other hand, if $\tilde{\alpha }>0$, then confidence starts to have more influence in the learning rate, with higher values of confidence having a stronger influence in the update rule. We define the prediction error vector

$$\delta_{t}=R_{t}-{\hat{R}}_{t}(\tilde{\theta })\cdot e^{\tilde{k}\cdot \text{cos}((\tilde{\theta }-\theta )-1)}$$ (28)

This definition implies that the reward distribution vector $\hat{R}$ is updated according to the location of the chosen stimulus $\tilde{\theta }$ smoothed by parameter $\tilde{k}$ and modulated by learning rate α*_t_*. Last, the reward distribution $\hat{R}$ is transformed to the resource gain function *g* via a normalization operation

$$g_{t+1}\leftarrow \frac{{\hat{R}}_{i,t}}{\sum_{i}{\hat{R}}_{t}}$$ (29)

Hence, this RL model has only three free parameters that are fitted to the observed data: ${\tilde{\alpha }}^{+}$, ${\tilde{\alpha }}^{-}$, and $\tilde{k}$. Vector ${\hat{R}}_{t}$ was discretized in steps of 2° from 0° to 178° in the orientation space.

### Model fits

For a given model *M*, we denote its set of parameters by a vector **Ψ**. The goal is to find the combination **Ψ** that maximized the probability of all of a single subject’s responses given the presented stimuli and the parameters. In this case, the log of the parameter likelihood function is

LL(Ψ;M)=log p( data ∣Ψ,model ) (30)

$$=\text{log}\;\prod_{i=1}^{N_{\text{trial}}}p(D_{i}|\theta_{n,l},\theta_{n,r},c_{n,l},c_{n,r},\Psi )$$ (31)

$$=\sum_{n}^{N_{\text{trials}}}\text{log}\;p(D_{i}|\theta_{n,l},\theta_{n,r},c_{n,l},c_{n,r},\Psi )$$ (32)

where θ and *c* are the orientation and contrast inputs for the left (l) and right (r) alternative, respectively, and *D_i_* is the mouse’s response on trial *i*. The implementation of the likelihood function was implemented using the mle2 function in the bbmle library implemented in the software package R. We typically performed an initial stage with 2000 randomly chosen initial parameter combinations. For each model *M*, we repeated this procedure three times, leading to log likelihood values that were typically within one point. We are therefore reasonably confident that we found the global maxima for each model. As a model comparison method, we use the Akaike information criterion (AIC) and the Bayesian information criterion.

### Poisson neural model

We investigated whether a more biologically relevant model of V1 function would also be able to capture characteristics of the rational inattention codes observed in the algorithmic model. An advantage of this implementational approach is that we can relate the metabolic cost directly to the expected number of spikes that the system generates to solve the decision problem.

The neural population consisted of Poisson neurons, each generating spikes *r* independently for a given input stimulus θ with probability

$$p(r|\theta )=\frac{e^{-f(\theta )}f{\left(\theta \right)}^{r}}{r!}$$ (33)

The tuning functions *f* of the neurons follow a bell-shaped activation pattern of the form

$$f_{n}(\theta )=g(\theta_{i})k(c)\text{exp}\;(\hat{\kappa }(\text{cos}\;(\theta_{i}-\theta )-1))+\Delta$$ (34)

where *g*() is a multiplicative gain partially determining the distribution of coding resources through the orientation space, *k*(*c*) is the multiplicative gain that the determines firing rate as a function of contrast, θ*_i_* is the preferred orientation of neuron *i*, and Δ is the base firing rate of a neuron at rest. $\hat{\kappa }$ controls the width of the tuning curve. The tuning curve width $\hat{\kappa }$ was estimated from the same study that we used to derive the prior distribution π(θ) (see Results and fig. S1) (*27*).

On the basis of this specification, the log likelihood function of population vector activity $\vec{r}$ can written as

$$\text{log}\;[p(\vec{r}|\theta )]=\sum_{n}^{N}\text{log}\;\frac{e^{-f_{n}(\theta )}f_{n}{\left(\theta \right)}^{r_{n}}}{r_{n}!}$$ (35)

To derive a posterior estimate $\hat{\theta }$, we assumed the BLS estimate. The BLS estimator can be approximated with discrete sums

$${\hat{\theta }}_{\text{BLS}}(\vec{r})\approx \frac{\sum_{n}\theta_{n}p(\vec{r}|\theta_{n})p(\theta_{n})\delta_{n}}{\sum_{n}p(\vec{r}|\theta_{n})p(\theta_{n})\delta_{n}}$$ (36)

in which θ*_n_* is a discrete set of stimulus values and δ*_n_* is the spacing between adjacent values. If θ*_n_* (the preferred stimuli of the neurons) is distributed in the stimulus space proportional to the prior distribution θ*_i_*, then $\delta_{n}\propto \frac{1}{p(\theta_{n})}$ (*38*, *46*); thus, the BLS estimator can be written as

$$\begin{array}{c} {\hat{\theta }}_{\text{BLS}}(\vec{r})\\ \approx \frac{\sum_{n}\theta_{n}p(\vec{r}|\theta_{n})}{\sum_{n}p(\vec{r}|\theta_{n})}\\ \approx \frac{\sum_{n}^{N}\theta_{n}\text{exp}\;(\sum_{m}^{M}r_{m}\text{log}\;f_{m}(\theta_{n})-\sum_{m}^{M}f_{m}(\theta_{n})-\sum_{m}^{M}\text{log}\;(r_{m}!))}{\sum_{n}^{N}\text{exp}\;(\sum_{m}^{M}r_{m}\text{log}\;f_{m}(\theta_{n})-\sum_{m}^{M}f_{m}(\theta_{n})-\sum_{m}^{M}\text{log}\;(r_{m}!))}\\ =\frac{\sum_{n}^{N}\theta_{n}\text{exp}\;(\sum_{m}^{M}r_{m}\text{log}\;f_{m}(\theta_{n})-\sum_{m}^{M}f_{m}(\theta_{n}))}{\sum_{n}^{N}\text{exp}\;(\sum_{m}^{M}r_{m}\text{log}\;f_{m}(\theta_{n})-\sum_{m}^{M}f_{m}(a_{n}))} \end{array}$$

where, in the last step, the last sum inside the exponential does not depend on the stimulus and can be dropped from the numerator and denominator.

Applying this model to our decision task, we assume that, for two inputs θ_l_ and θ_r_ in any given trial, the model generates population activity ${\vec{r}}_{l}$ and ${\vec{r}}_{r}$ independently for each input, which leads to the computation of estimates ${\hat{\theta }}_{l}$ and ${\hat{\theta }}_{r}$. Then, a choice is made on the basis of the decision rule of our task.

The goal of the neural network is to find a balance between model performance (i.e., reward intake) and the metabolic costs associated to neural activity in the network. More formally, we need to find the optimal resource gain function *g* and a maximum activity *r*_max_ such that

$$\underset{g,k_{\text{max}}}{\text{max}}\;E[\text{reward}|\Omega ,k(c),\sigma_{\text{late}},\pi (\theta )]-\eta E[\text{spikes}]$$ (37)

where, in this case, η corresponds to unit cost per spike generated by the network.

## Notes

View/request a protocol for this paper from *Bio-protocol*.
