# Supplementary material for: Rational inattention in mice
Source: Sci Adv. 2022 Mar 4;8(9):eabj8935. doi: 10.1126/sciadv.abj8935 (PMC8896787; doi:10.1126/sciadv.abj8935)
Supplement: Supplementary file 1 — Figs. S1 to S13 Tables S1 and S2 [file sciadv.abj8935_sm.pdf]

Science Advances
AAAS

Supplementary Materials for

Rational inattention in mice

Nikola Grujic, Jeroen Brus, Denis Burdakov*, Rafael Polania*

*Corresponding author. Email: rafael.polania@hest.ethz.ch (R.P.); denis.burdakov@hest.ethz.ch (D.B.)

Published 4 March 2022, *Sci. Adv.* **8**, eabj8935 (2022)
DOI: 10.1126/sciadv.abj8935

This PDF file includes:

Figs. S1 to S13
Tables S1 and S2

Supplementary Figure 1

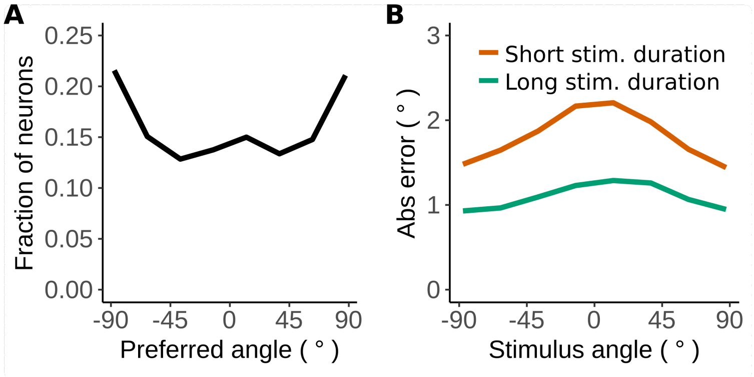

Mouse V1 has more coding resources at the horizontal angles. Stringer et al. (28) recorded multi-plane two-photon calcium images from the primary visual cortex of awake mice. Stimuli were static gratings rotated at random orientations. (A) Each neuron has a tuning curve with a strongest response to a preferred orientation. The distribution of preferred orientations across cells shows more neurons prefer horizontal angles. (B) Stringer et al. used a linear decoder to estimate the stimulus orientation from neuronal activity. They found that coding error is higher for the vertical angles. When the stimulus duration was shortened (100 ms (red) vs. 750 ms (green)) the decoding error of the linear decoder goes up. The results presented in A) and B) are indicative that mice are more exposed to horizontal than to vertical orientations in the natural world. To simulate the natural exposure over orientations the prior distribution $\pi(\theta)$ used in our behavioral task is also higher for horizontal than vertical orientations. This allows us to study the effects of changing reward-stimulus contingencies on the coding strategies.

Supplementary Figure 2

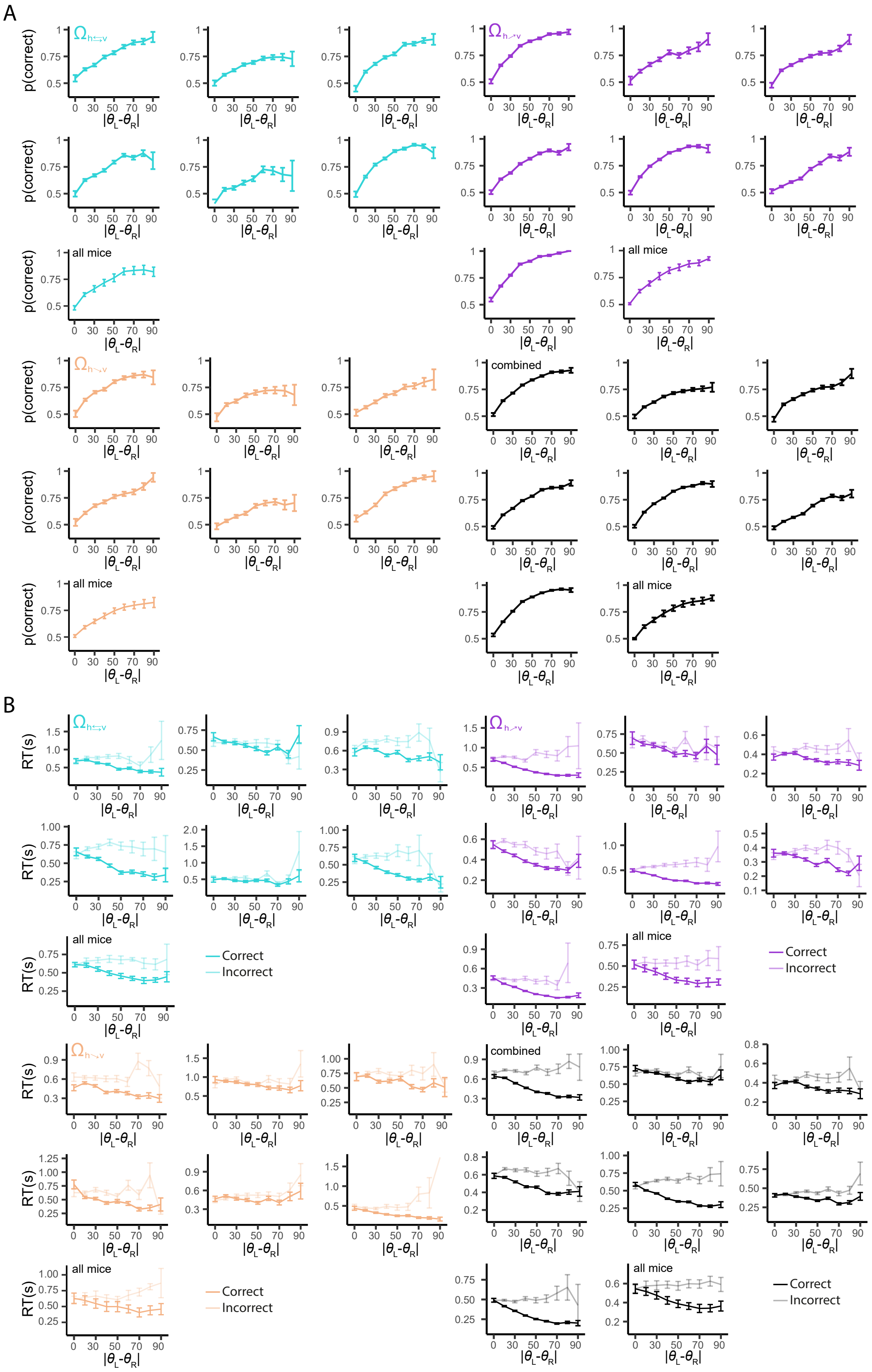

Psychometrics per mouse. (A) Each panel shows the probability of correct choice at different absolute angle differences for one mouse, with the final panel showing combined data within the condition. Panels with black lines show combined reward environments, and individual environments are shown color coded and marked in top left corner. (B) Each panel shows the

average reaction times (RTs) at different absolute angle differences. The transparent lines are for incorrect and full lines for correct trials. All error bars are standard errors.

Supplementary Figure 3

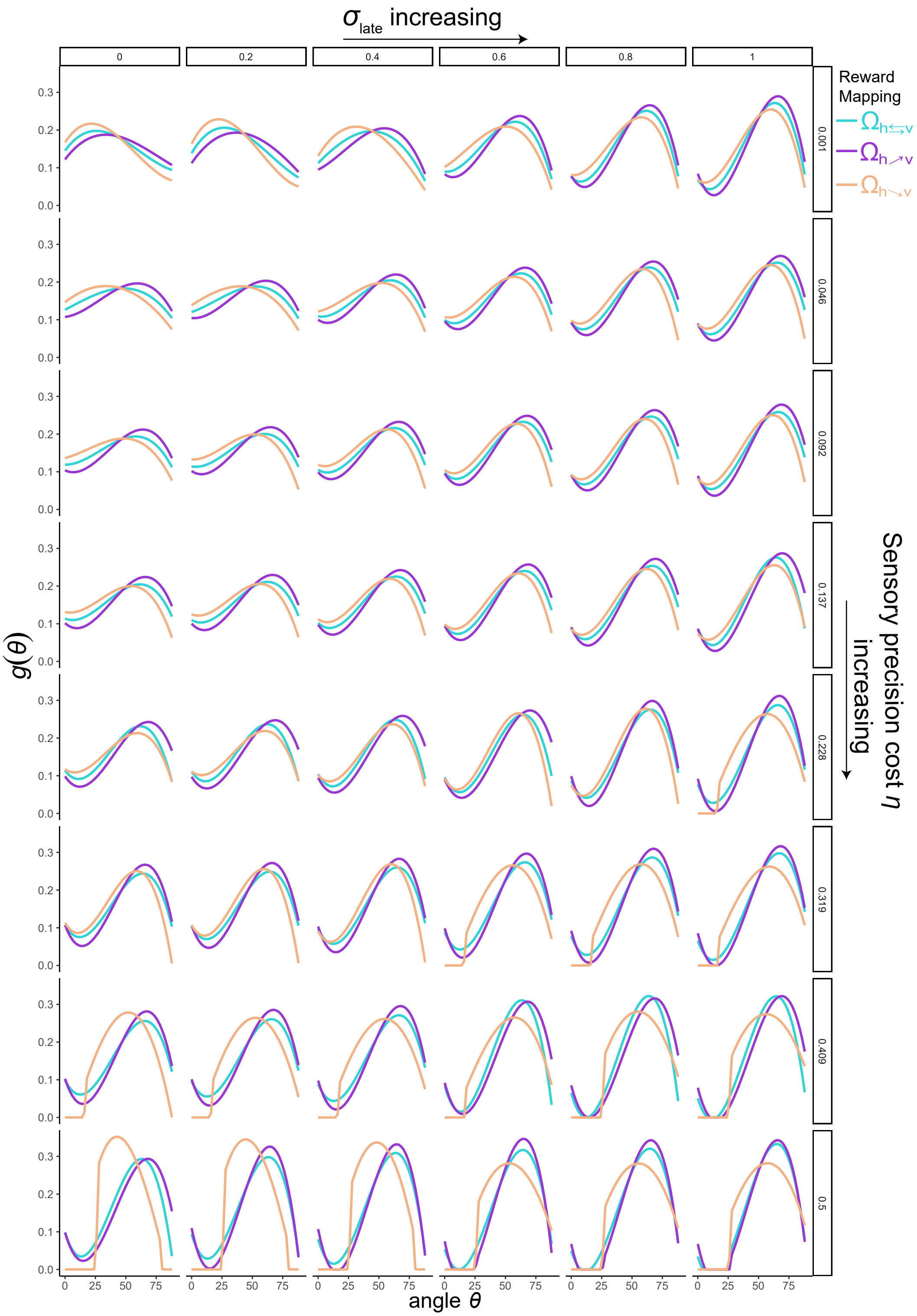

Optimal $g(\theta)$ depends on precision costs and noise. The way in which the system should invest in sensory encoding for a particular portion of the stimulus space depends on both precision costs η (increasing from top to bottom) and late noise σ_{late} (increasing from left to right). An interesting property of the rational inattention model is that as both η and σ_{late} increase, the fewer resources the system invests on regions of the stimulus space with higher density. This demonstrates important differences between solutions of efficient coding that that assume low levels of noise in the encoder (e.g., top left panel) and solutions where the low-noise regime is dropped.

Supplementary Figure 4

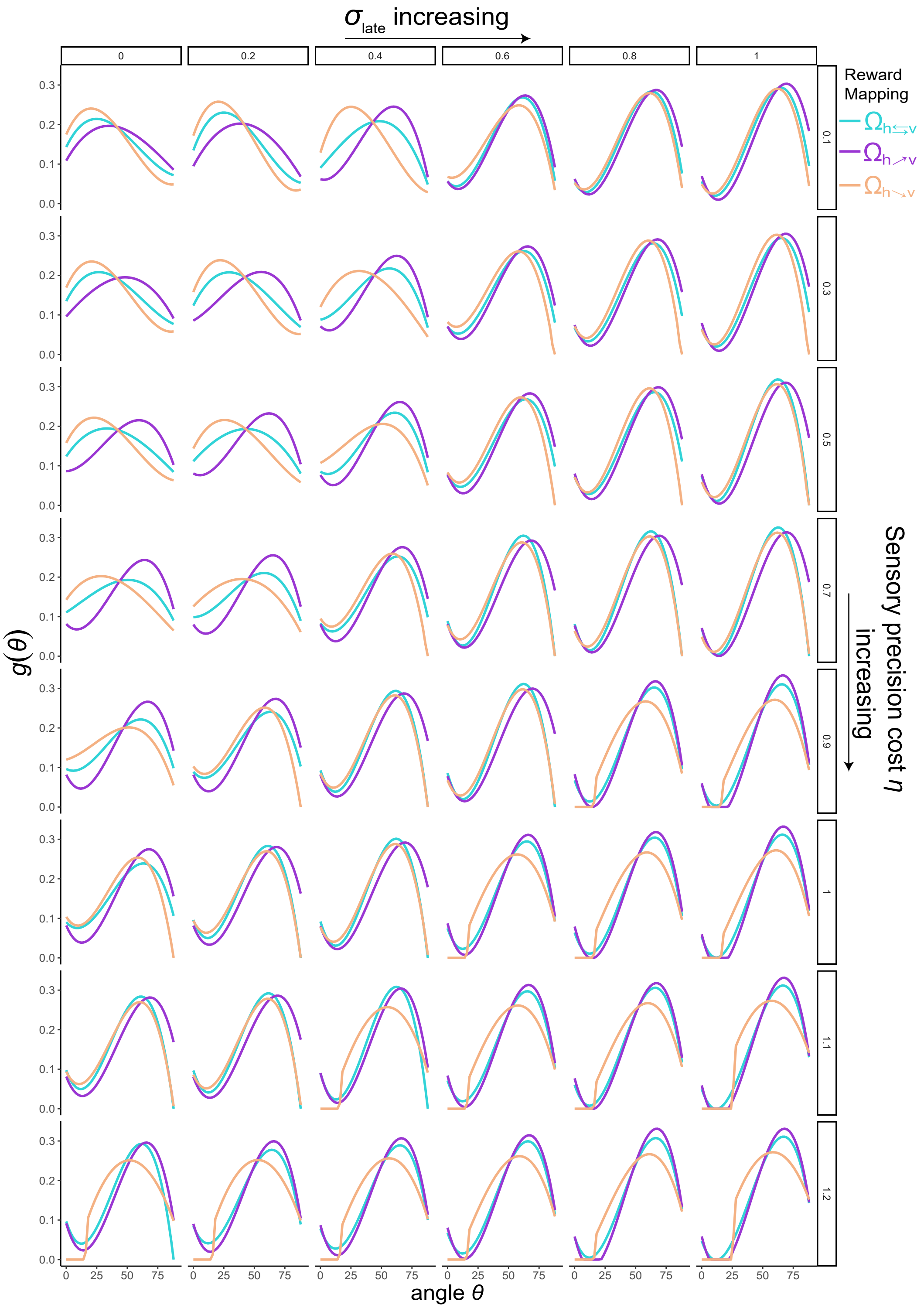

Optimal $g(\theta)$ using entropy as cost function. This figure shows similar information to Supplementary Figure 3, with the difference that now the cost function is the expected reduction in entropy $\mathbb{E}[H(\theta \mid m)]$. While there are slight differences in the solutions for $g(\theta)$, these are in general qualitatively similar to the solutions for using precision as cost function. The reason is that both cost functions increase with the use of more resources. However, the relationship between expected precision and entropy reduction is not linear (see Supplementary Figure 5).

Supplementary Figure 5

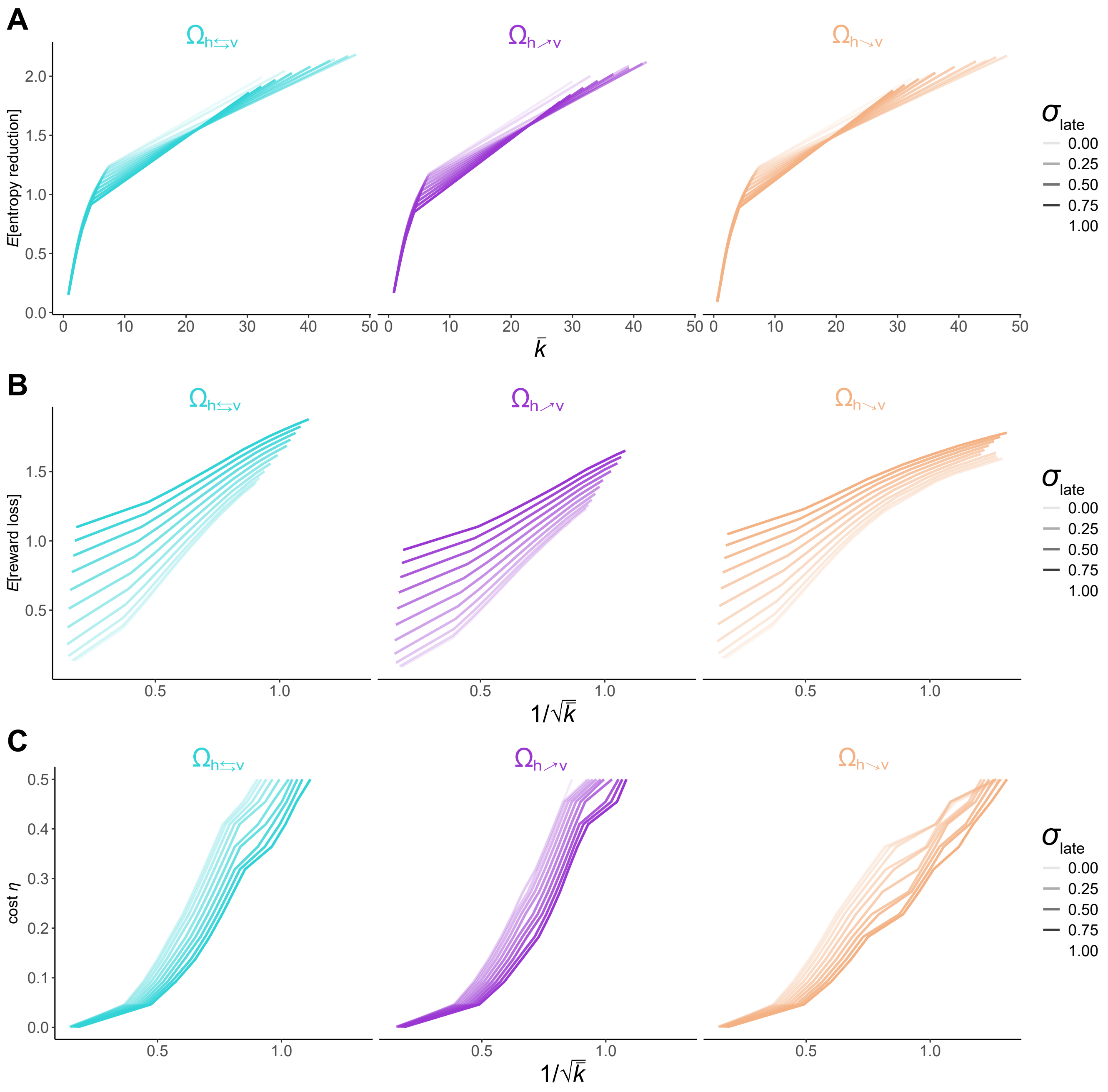

Relationship between average precision, entropy reduction, reward loss, and cost. (A) Relationship between average precision $\bar{k}$ and entropy reduction $\mathbb{E}[H(\theta \mid m)]$ for the rational inattention model optimized for a linear precision cost for each environment Ω (left, middle, and right panels), and for different levels of late noise σ_{late} (different levels of line transparency). The results of this analysis show that the relationship between these two metrics is non-linear, showing signatures of concavity. (B) Relationship between the inverted squared root of average precision $1/\sqrt{\bar{k}}$ and expected reward loss for the rational inattention model optimized for linear precision cost for each environment Ω (left, middle, and right panels), and for different levels of late noise σ_{late} (different levels of line transparency). As expected, the higher $1/\sqrt{\bar{k}}$ and σ_{late}, the higher the expected reward loss. (C) Relationship between $1/\sqrt{\bar{k}}$ and linear precision cost for linear precision cost η for each environment Ω (left, middle, and right panels), and for different levels of late noise σ_{late} (different levels of line transparency). As expected, the higher $1/\sqrt{\bar{k}}$ (i.e., smaller invested precision) the higher cost. Also note that for a fixed cost level, the smaller the level of late noise σ_{late}, the lower the precision that the organism/system should invest.

Supplementary Figure 6

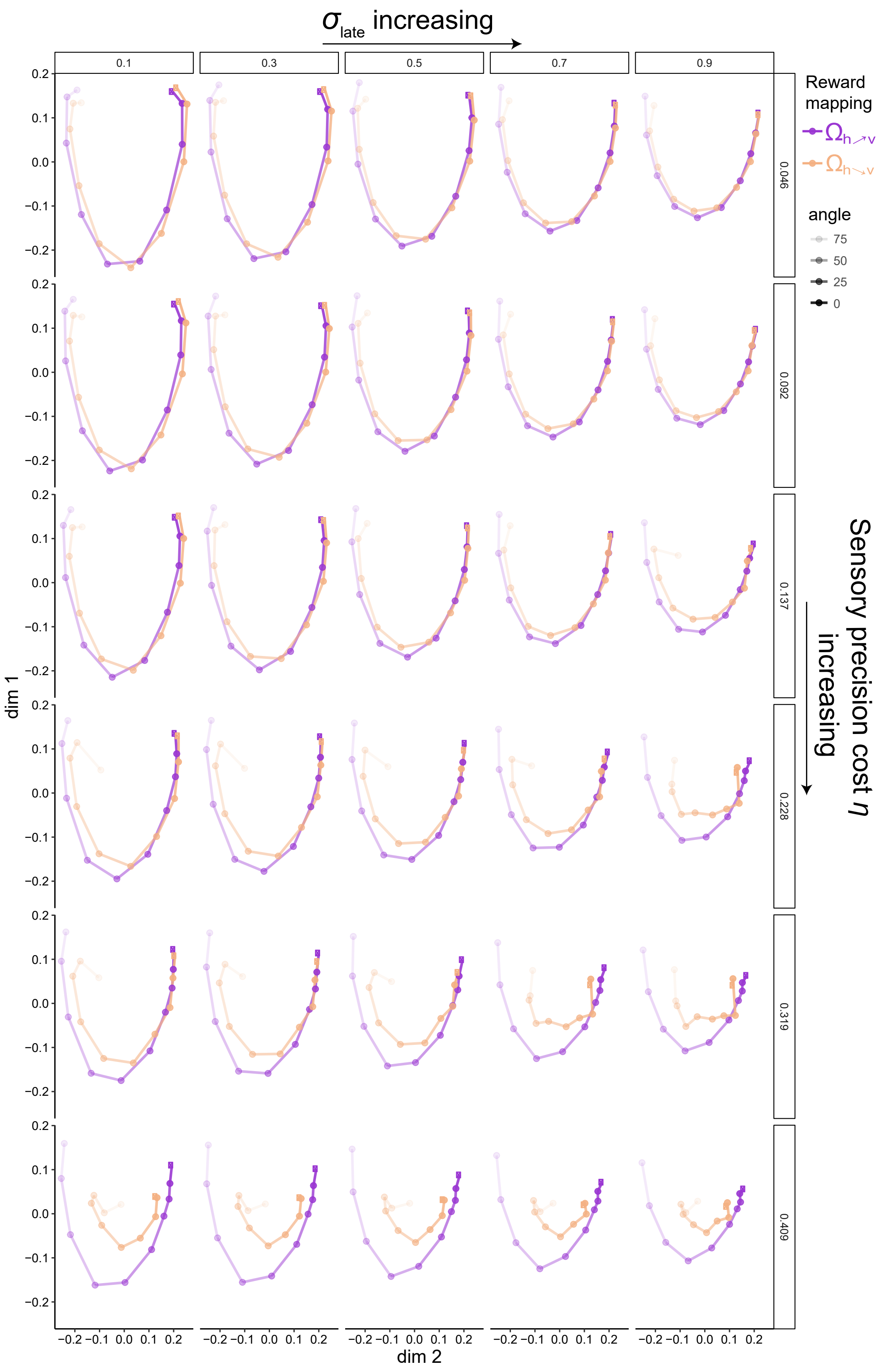

Geometric analyses of psychophysical performance of the rational inattention model allowing sensory resources g to be adaptive across the sensory space. In order to have a better overview of predicted discriminability performance across all pairwise combinations of input stimuli as a function of precision cost η and downstream noise σ_{late}, we implemented a

multidimensional scaling (MDS) analysis. For clarity in the presentation of these analyses, we considered only environments $\Omega_{R:h\searrow v}$ and $\Omega_{R:h\nearrow v}$, which are diametrically orthogonal in terms of stimulus-reward mappings. The introduction of MDS analyses is appealing because it provides an intuitive interpretation of behavioral performance. Here, each dot represents an input stimulus (where each angle in is represented with a different transparency level). The distance between a pair of nodes in the manifold then represents degree of discriminability between the two angles. In our task the MDS shows various important features of psychophysical performance: (i) The higher the sensory precision cost η and downstream noise σ_{late} the shorter the distance between dots, thus indicating lower levels of overall discriminability. (ii) In enviroment $\Omega_{R:h\nearrow v}$ discriminability at horizontal angles is smaller relative to environment $\Omega_{R:h\searrow v}$, and vice-versa for vertical angles. (iii) For a given level of sensory precision cost η and downstream noise σ_{late} psychophysical performance is better in environment $\Omega_{R:h\nearrow v}$ relative to $\Omega_{R:h\searrow v}$. This prediction emerges in the rational inattention framework because of the resource constraints and inference process considered here. For a given level of sensory and decision precision, reward expectation in environment $\Omega_{R:h\nearrow v}$ is higher. Thus our rational inattention model generates a set of testable predictions that can be verified with the empirical data.

Supplementary Figure 7

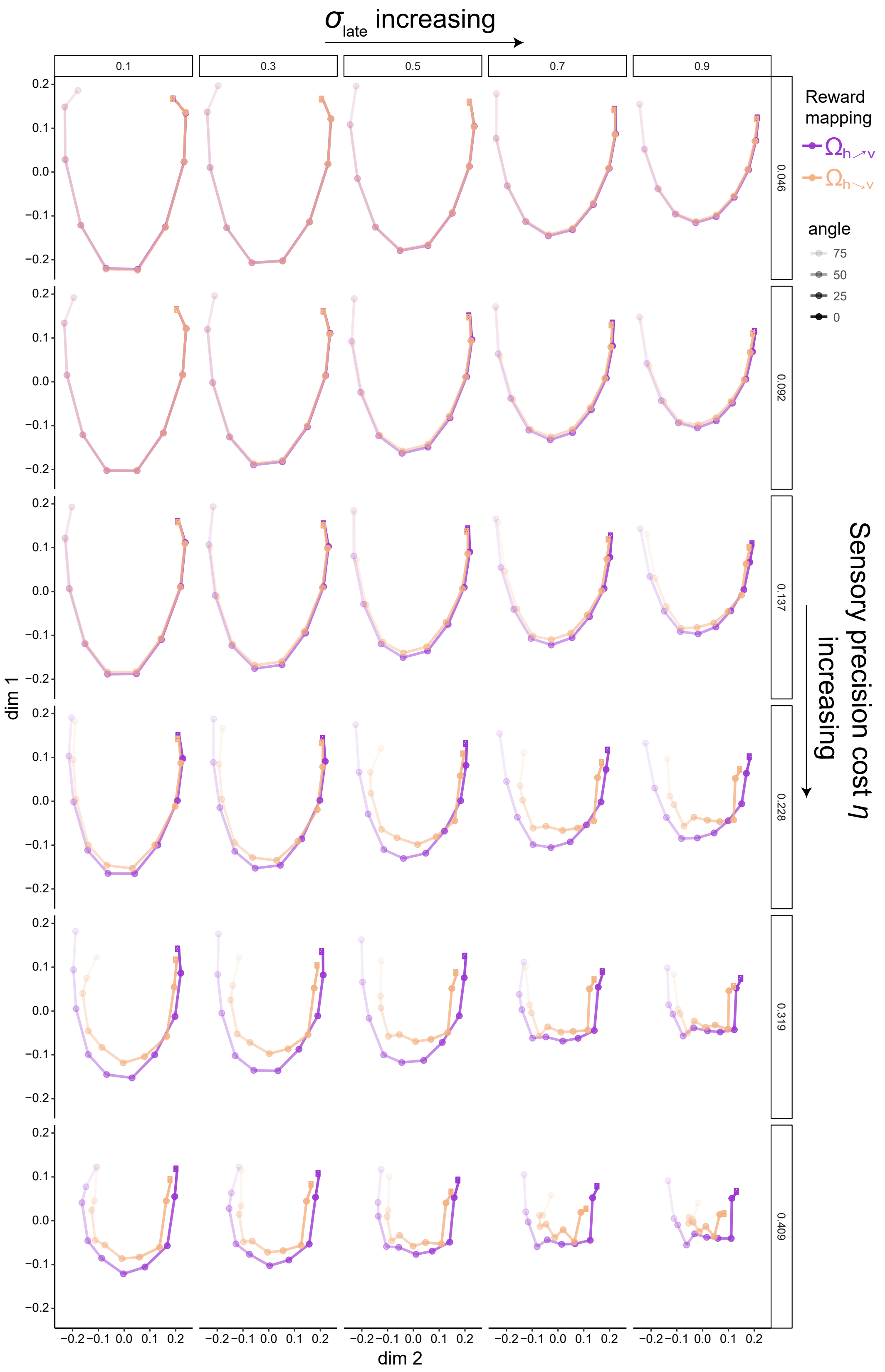

Geometric analyses of psychophysical performance of the rational inattention model assuming uniform resource gain function g **across the sensory space.** Here we conducted MDS analyses in the same way as conducted in Supplementary

Figure 6, but this time assuming a uniform resource gain function g across the sensory space. While for a given level of sensory precision cost η and downstream noise σ_{late} psychophysical performance is better in environment $\Omega_{R:h \nearrow v}$ relative to $\Omega_{R:h \searrow v}$, and thus similar to the prediction presented in Supplementary Figure 6 (a general prediction of the rational inattention framework), the degree discriminabilty is relatively constant across the sensory space. Additionally, for the same levels of sensory precision cost η and downstream noise σ_{late}, the degree of discriminability is larger for the variable gain g rational inattention model (Supplementary Figure 6) relative to the uniform gain g model (this figure). This is qualitatively evident by comparing the overall distance between the nodes of the manifold across Figures S6 and S7. Thus the two types of rational inattention models provide distinct qualitative features that can be qualitatively compared against empirical data.

Supplementary Figure 8

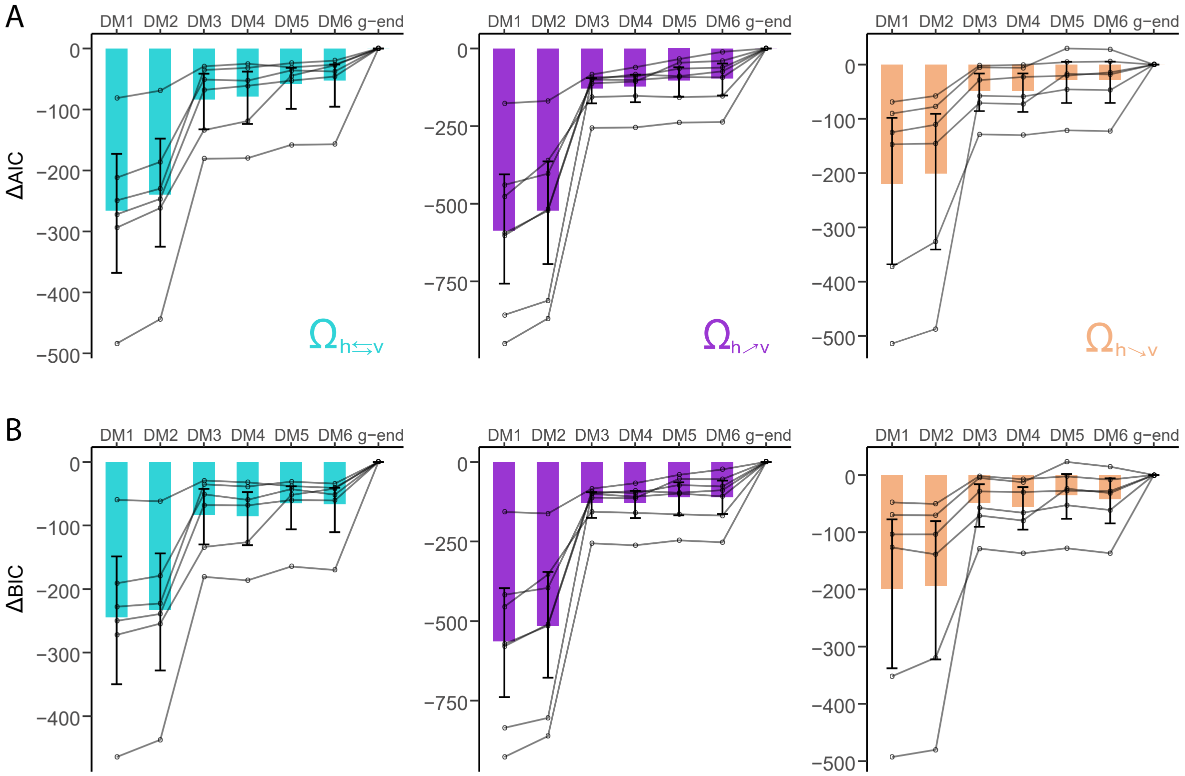

AIC and BIC comparisons for all models. To further validate the efficacy of our rational inattention model, we compared it to a few other models. In addition to the models shown in the main text (DM1 to DM3), we investigated three new models with additional parameters. These models were the same as DM3 (see Methods) with the following changes: DM4 included separate parameters for lapse for each side, instead of just one, as in previous models. DM5 contained a parameter for reward size received in the previous trial. DM6 contained both a separate parameter for each side lapses, as well as for reward size in the previous trial. (A) Difference in AIC between the rational inattention model (g-end) and all other descriptive models (DMs), from the simplest (DM1) to the most complex DM considered here (DM6). (B) Same as panel A, but quantitative model comparison is based on the Bayesian Information Criteria (BIC).

Supplementary Figure 9

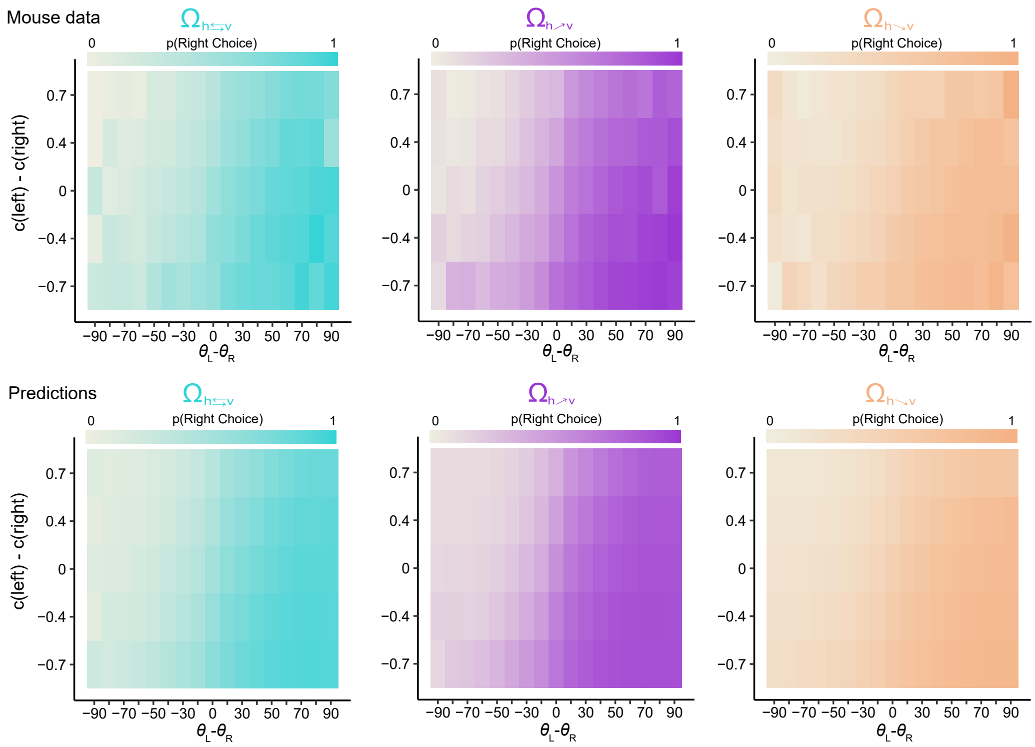

Mouse data and rational inattentive model predictions for difference in contrast levels. Heatplots for each reward environment (denoted on top of heatplot and color coded) show probability of choosing the right side stimulus at each contrast difference in the study vs. the angle difference (trial difficulty). Top three plots show real mouse data and bottom three show predictions of the static model. The contrast differences between two stimuli that were 0.4 and 0.3 were grouped together for the purpose of this plot, and are marked 0.4 on the y axis. Preference for higher contrast side stimuli as a function of difficulty, which could be interpreted as risk aversiveness, is observed in these plots, as well as the ability of our model to predict it.

Supplementary Figure 10

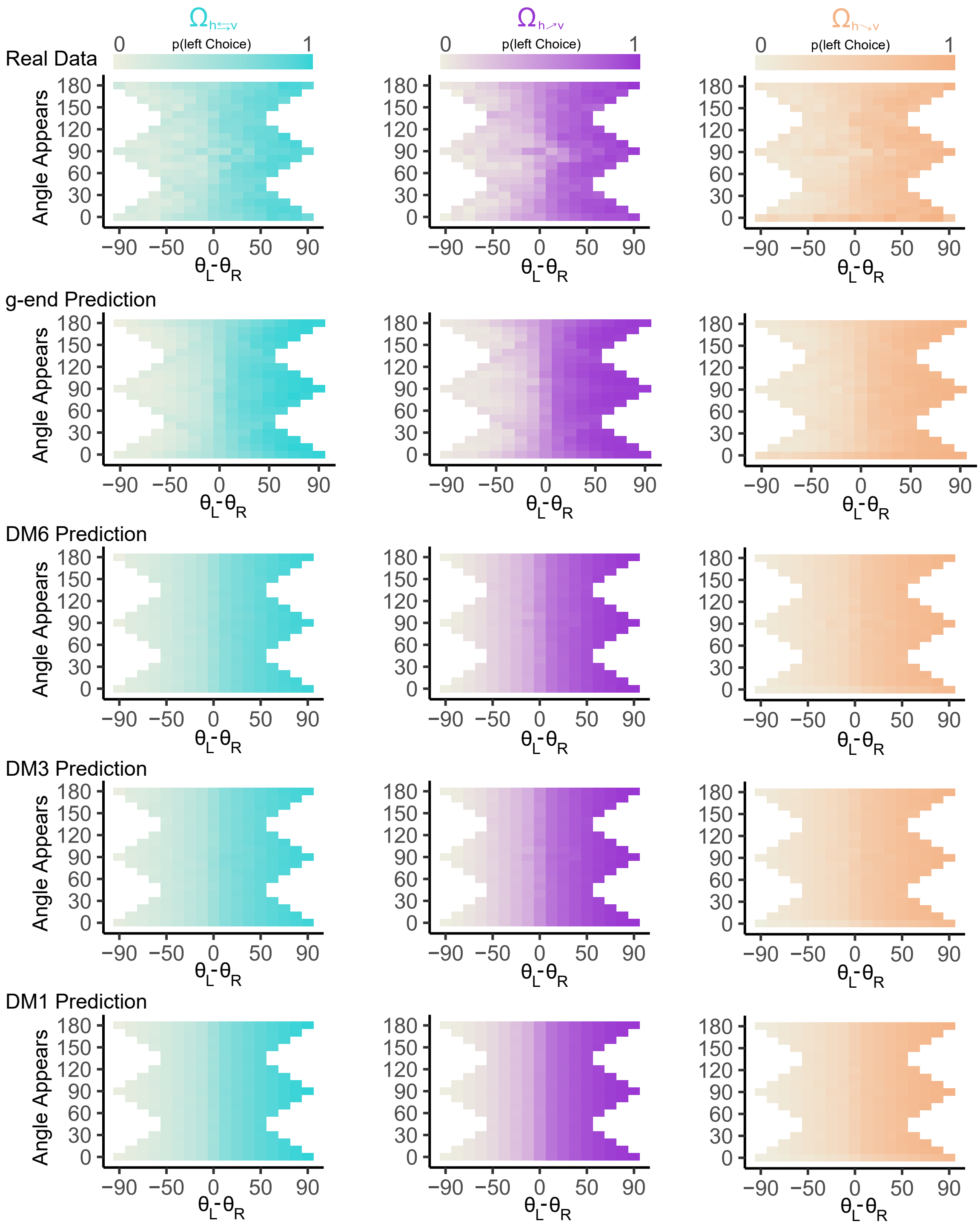

Real and predicted data for different models. Each panel shows a heatplot of probability of choosing the left side stimulus. An angle appearing in a trial is on the y axis, and difference in angle is on the x axis. Heatplots are shown for real data, some of the model predictions, and for all reward environments.

Supplementary Figure 11

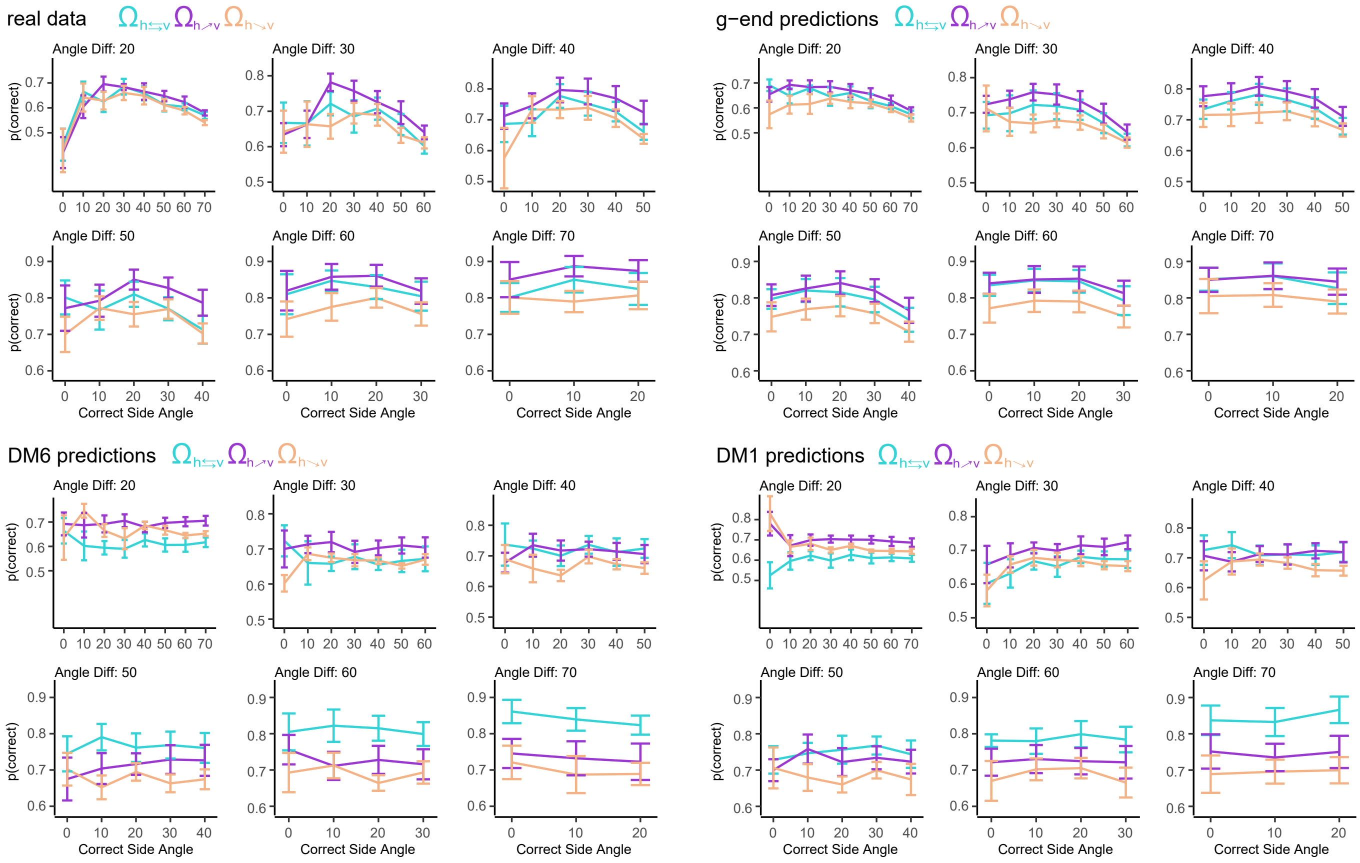

Real and predicted choice at different angles. Each panel shows the probability of correct choice on a trial, given a certain absolute correct-side angle. Angle difference is fixed for each panel and indicated in the top left corner. Data is color coded for reward environment. We show real mouse data in the top left panels. Top right are predictions from the rational inattention model. Bottom left are predictions from DM6 (the most complicated descriptive model) and bottom right from DM1 (the simplest descriptive model).

Supplementary Figure 12

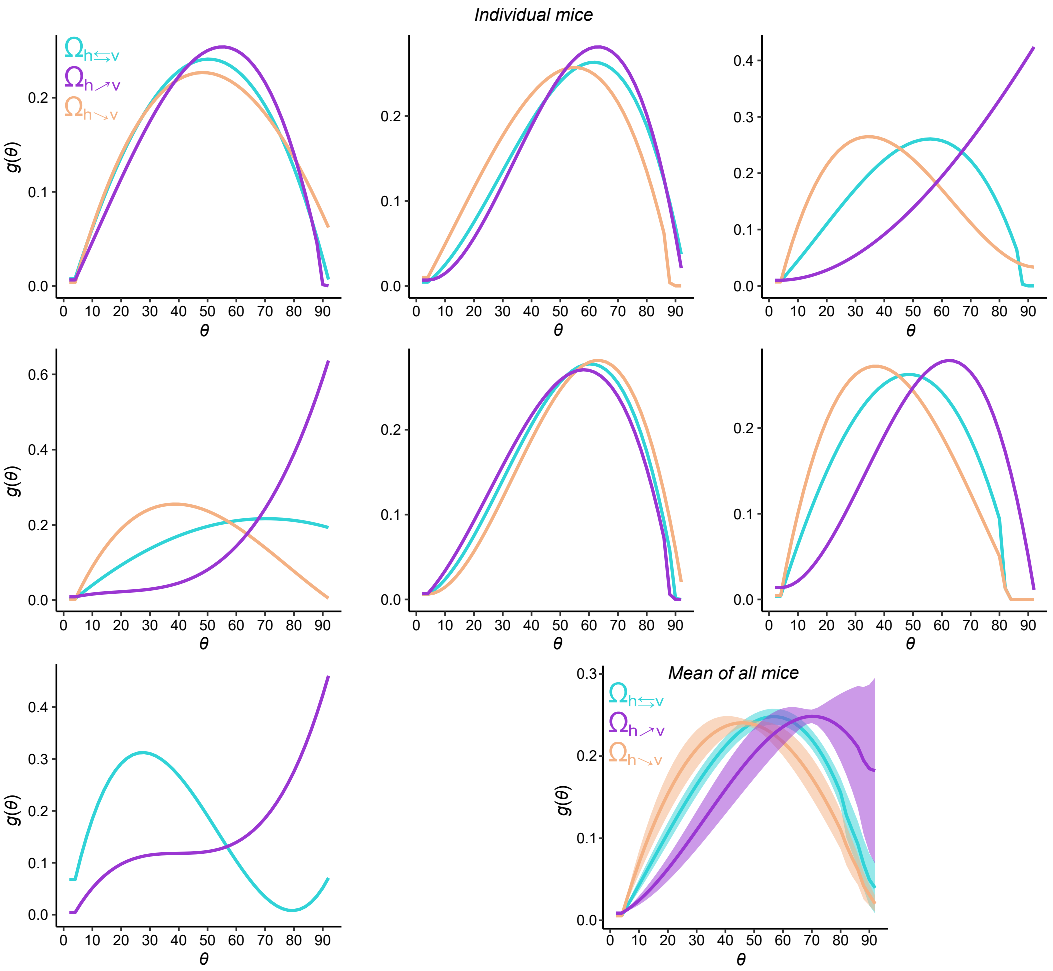

Individual animals show adaptation to different reward environments. Resource allocation functions $g(\theta)$ for each reward environment Ω and each individual mouse. Bottom right plot shows the means of all mice that had all reward environments (top two rows). Shaded areas show standard 95% bootstrap CIs. Individual animals show the trend of increasing the resources allocated in areas of stimulus space with higher reward sizes. Gain shifts to more horizontal orientations in $\Omega_{R:h\searrow v}$, as more reward is dedicated to that portion of stimulus space in this reward condition. The effect is the opposite for $\Omega_{R:h\nearrow v}$, and in between those two is the $\Omega_{R:h\rightleftarrows v}$, as would be intuitive looking at reward mappings in Fig. 1E.

Supplementary Figure 13

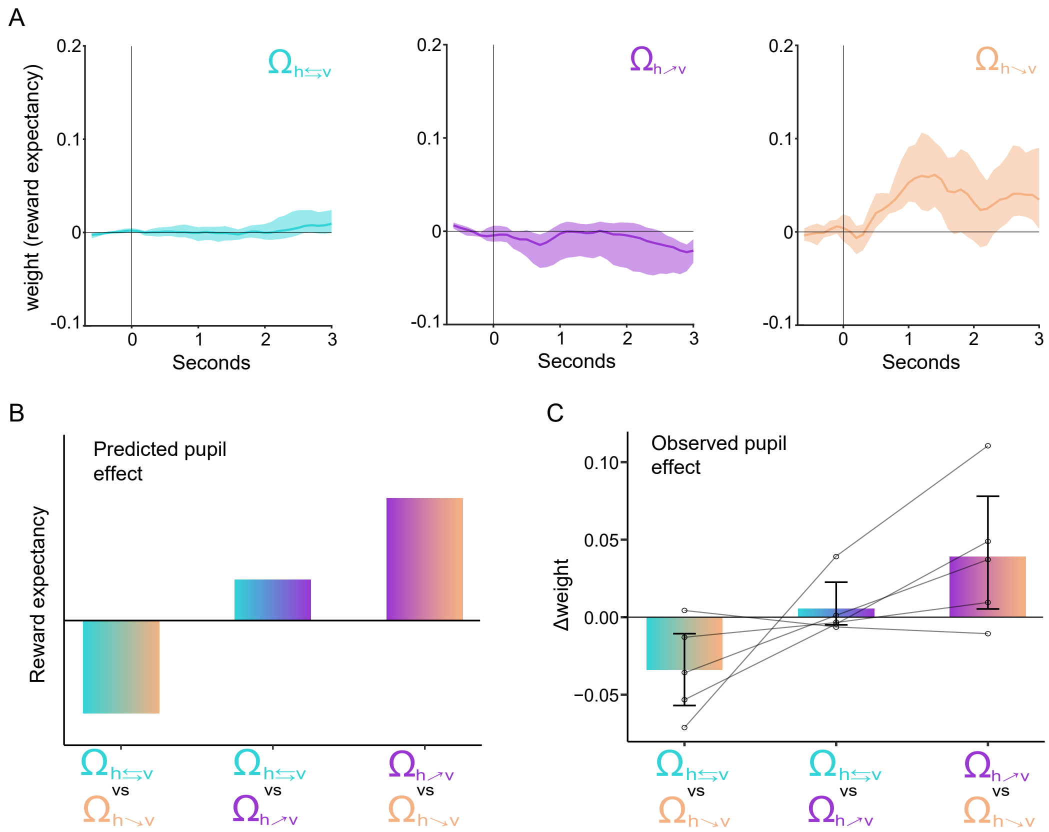

Reward expectancy effect on pupil. (A) Weights of expected reward effect on phasic pupil response over time for each reward environment Ω. Aligned to stimulus onset. Shaded areas show 95% bootstrap CIs. (B) Predicted reward environment pairwise differences in effect of reward expectancy on phasic pupil response. (C) Observed pairwise reward environment differences in weights of expected reward effect on phasic pupil response. Averaged from stimulus onset to 3 seconds post onset. Error bars show 95% CIs and lines show differences in individual mice.

Supplementary Table 1

	Correct side angle (degrees)									
Reward Mappings	0	10	20	30	40	50	60	70	80	90
$\Omega_{R:h\nearrow v}$, µL	1	1.8	2.6	3.3	4.1	4.9	5.7	6.4	7.2	8
$\Omega_{R:h\searrow v}$, µL	8	7.2	6.4	5.7	4.9	4.1	3.3	2.6	1.8	1
$\Omega_{R:h\rightleftarrows v}$, µL	5	5	5	5	5	5	5	5	5	5

Stimulus-reward mapping. The amounts of milkshake dispensed at a given correct side angle in a given reward environment Ω. In $\Omega_{R:h\nearrow v}$, reward size increases from horizontal to vertical. The opposite is true for the $\Omega_{R:h\searrow v}$ mapping, whereas same size of reward is assigned to all angles in the $\Omega_{R:h\rightleftarrows v}$ reward mapping condition.

Supplementary Table 2

Reward Mapping	Model	σ1	σ2	σ3	bias	lapse	previous choice	contrast difference	previous reward	lapse left	lapse right
$\Omega_{h\leftrightarrows v}$	DM1	1.36 ± 0.09	/	/	0.20 ± 0.42	0.09 ± 0.05	0.17 ± 0.11	/	/	/	/
	DM2	1.85 ± 0.17	1.12 ± 0.10	1.12 ± 0.11	0.21 ± 0.41	0.10 ± 0.06	0.19 ± 0.13	/	/	/	/
	DM3	1.77 ± 0.12	1.15 ± 0.09	1.11 ± 0.09	0.25 ± 0.45	0.08 ± 0.05	0.18 ± 0.11	0.08 ± 0.04	/	/	/
	DM4	1.77 ± 0.13	1.14 ± 0.06	1.08 ± 0.08	0.20 ± 0.41	/	0.15 ± 0.10	-0.38 ± 0.02	/	0.11 ± 0.06	0.06 ± 0.03
	DM5	1.85 ± 0.16	1.20 ± 0.09	1.17 ± 0.09	0.23 ± 0.45	0.06 ± 0.03	0.30 ± 0.07	0.06 ± 0.03	0.06 ± 0.04	/	/
	DM6	1.86 ± 0.20	1.19 ± 0.08	1.14 ± 0.09	0.18 ± 0.41	/	0.30 ± 0.08	-0.36 ± 0.02	-0.02 ± 0.03	0.09 ± 0.04	0.03 ± 0.02

Reward Mapping	Model	σ1	σ2	σ3	bias	lapse	previous choice	contrast difference	previous reward	lapse left	lapse right
$\Omega_{h\searrow v}$	DM1	1.54 ± 0.14	/	/	0.03 ± 0.46	0.09 ± 0.03	0.18 ± 0.09	/	/	/	/
	DM2	1.95 ± 0.19	1.31 ± 0.13	1.39 ± 0.16	0.03 ± 0.47	0.08 ± 0.03	0.18 ± 0.09	/	/	/	/
	DM3	1.92 ± 0.19	1.32 ± 0.13	1.38 ± 0.15	0.03 ± 0.47	0.07 ± 0.04	0.18 ± 0.10	0.07 ± 0.04	/	/	/
	DM4	1.94 ± 0.18	1.33 ± 0.13	1.40 ± 0.15	-0.03 ± 0.42	/	0.18 ± 0.10	-0.36 ± 0.04	/	0.09 ± 0.04	0.05 ± 0.02
	DM5	1.86 ± 0.16	1.27 ± 0.11	1.35 ± 0.13	0.02 ± 0.47	0.08 ± 0.04	-0.16 ± 0.09	0.08 ± 0.04	0.08 ± 0.01	/	/
	DM6	1.85 ± 0.15	1.27 ± 0.10	1.34 ± 0.12	-0.02 ± 0.42	/	-0.15 ± 0.09	-0.38 ± 0.04	0.08 ± 0.02	0.11 ± 0.04	0.07 ± 0.04

Reward Mapping	Model	σ1	σ2	σ3	bias	lapse	previous choice	contrast difference	previous reward	lapse left	lapse right
$\Omega_{h\nearrow v}$	DM1	0.96 ± 0.05	/	/	0.25 ± 0.38	0.17 ± 0.06	0.08 ± 0.06	/	/	/	/
	DM2	1.15 ± 0.07	0.80 ± 0.04	0.69 ± 0.06	0.35 ± 0.43	0.20 ± 0.07	0.08 ± 0.06	/	/	/	/
	DM3	1.29 ± 0.10	0.95 ± 0.08	0.84 ± 0.06	0.22 ± 0.40	0.13 ± 0.05	0.06 ± 0.05	0.13 ± 0.03	/	/	/
	DM4	1.40 ± 0.12	1.05 ± 0.09	0.92 ± 0.07	0.14 ± 0.35	/	0.04 ± 0.04	-0.43 ± 0.03	/	0.10 ± 0.05	0.04 ± 0.02
	DM5	1.33 ± 0.10	0.99 ± 0.09	0.90 ± 0.08	0.20 ± 0.40	0.11 ± 0.04	0.08 ± 0.09	0.11 ± 0.02	0.11 ± 0.03	/	/
	DM6	1.41 ± 0.12	1.05 ± 0.10	0.93 ± 0.07	0.15 ± 0.35	/	0.09 ± 0.09	-0.43 ± 0.03	0.00 ± 0.02	0.10 ± 0.05	0.04 ± 0.02

Fitted parameters of all descriptive models (DMs) Overview of parameters in each of the models, and parameter coefficients for each reward environment. Each value is a mean across mice ± standard error (SE).
